# Synergistic Regulation Mechanism of Anti-Dispersion and Flowability of Alkali-Activated Slag Underwater Non-Dispersible Slurry

**DOI:** 10.3390/ma19173633

**Published:** 2026-08-26

**Authors:** Shengnan Xu, Fumin Li, Li Zhang, Yangmei Zhou, Yanpeng Zhao, Yongsheng Ji

**Affiliations:** 1 Jiangsu Key Laboratory Environmental Impact and Structural Safety in Engineering, China University of Mining and Technology, Xuzhou 221008, China; yangmeizhou227@163.com (Y.Z.); jiyongsheng@cumt.edu.cn (Y.J.); 2 Department of Architecture and Transportation Engineering, Jiangsu College of Engineering and Technology, Nantong 226006, China; 3 Nanjing Construction Engineering Quality and Safety Supervision Station, Nanjing 210007, China

**Keywords:** alkali-activated slag, HPMC, PAM, flowability, anti-dispersion

## Abstract

The trade-off between flowability and anti-dispersion properties of alkali-activated slag slurry in underwater environments represents a key technical bottleneck limiting their application in marine underwater engineering. In this study, granulated blast furnace slag (GGBS) was used as the raw material, with the modulus of liquid sodium silicate adjusted by NaOH serving as the alkali activator, and hydroxypropyl methylcellulose (HPMC) and polyacrylamide (PAM) selected as anti-dispersion agents. This study systematically investigates the synergistic regulation mechanisms of the anti-dispersion agents’ type, dosage, and activator on the anti-dispersion properties and rheological behavior of alkali-activated slag slurry, and revealed the evolution mechanisms of the microstructure of the hardened slurry through X-ray diffraction (XRD), Fourier transform infrared spectroscopy (FT-IR), scanning electron microscopy (SEM) and mercury intrusion porosimetry (MIP) analysis. The results indicate that the activator is the key factor in regulating the various properties of the slurry, and the combination of PAM and HPMC produces a significant synergistic effect. At the optimal formulation (modulus of 1.0, total blended anti-dispersion agent content of 1%, and a mass ratio of PAM to HPMC of 1:1), the slurry exhibited a wet loss rate of 38.45%, a solid retention rate of 89.98%, a flow value of 195 mm, and a 28-day compressive strength of 41.62 MPa, achieving an optimal balance between anti-dispersion performance and workability.

## 1. Introduction

As the strategy for developing marine resources continues to advance, the demand for underwater infrastructure—such as cross-sea bridges, undersea tunnels, and offshore wind farm foundations—is growing, placing higher demands on underwater concrete construction technology [1,2,3,4]. Traditional underwater concrete construction methods, such as the cofferdam method and the pipe method [5], are characterized by complex processes and high costs. Furthermore, during underwater pouring, the scouring and dilution effects of water flow can easily cause segregation in fresh concrete [6,7], leading to quality defects such as insufficient strength and reduced durability in the cured concrete. These defects severely compromise the structural safety and service life of underwater structures.

To improve the underwater anti-dispersion properties of concrete, water-soluble polymer anti-dispersion agents have been introduced. By increasing the slurry viscosity and particle cohesion, this has led to the development of non-dispersible concrete technology [8]. Polyacrylamide (PAM) and hydroxypropyl methylcellulose (HPMC), as two typical water-soluble polymer anti-dispersion agents [9,10], have been widely used in this field.

Studies by Moon [8] and Guo [11] have shown that when HPMC is added, the hydroxyl groups and ether bonds in its molecular chains form hydrogen bonds with water, reducing the free water content in the slurry and thereby effectively improving its resistance to dispersion; even a 0.5 wt% addition is sufficient to provide a significant anti-dispersion effect; however, excessive addition can have a negative impact on workability and mechanical properties [12]. By adsorbing onto particle surfaces and forming bridging connections between molecular chains, PAM can effectively enhance the dispersion resistance of cement paste; furthermore, dispersion resistance continues to increase as the dosage increases [13]. However, this is accompanied by an increase in problems such as reduced liquidity and delayed hydration [5].

Although the aforementioned anti-dispersion agents have achieved some success in traditional silicate cement systems, practical applications still face issues such as poor compatibility between anti-dispersion agents and water-reducing agents, excessive loss of slurry workability over time, and significantly prolonged setting times [14,15], which severely limit the construction efficiency and engineering adaptability of underwater non-dispersive concrete. Therefore, the search for new cementitious material systems to replace traditional Portland cement has become a key direction for overcoming the limitations of existing technologies.

Alkali-activated slag, as a new type of low-carbon cementitious material, is characterized by excellent mechanical properties, rapid setting, a fine and dense pore structure, and superior impermeability. Additionally, the alkali activator possesses a certain degree of viscosity; when used in conjunction with an anti-dispersant, it can further enhance the slurry’s viscosity, demonstrating good application potential in underwater engineering projects.

However, the application of alkali-activated slag slurry in underwater engineering still faces the core scientific challenge of the trade-off between anti-dispersion properties and workability [16,17]. Although the addition of anti-dispersants can significantly enhance the underwater anti-dispersion performance of alkali-activated slag slurry, it also causes a sharp increase in slurry viscosity and a severe decline in workability, posing challenges to the constructability of underwater construction [17,18]. Bai [19] found that when HPMC was added to alkali-activated slag, both the yield stress and plastic viscosity of the slurry increased, the hydration process was delayed, porosity increased, and strength decreased. Yu [20] pointed out that the network structure formed by PAM and water through hydrogen bonding increased the shear stress and viscosity of the slurry, improving its resistance to dispersion but at the expense of flowability [20]. Sun [21] found that increasing the modulus of the activator can refine the particle size distribution of the slurry, reduce the volume of harmful capillaries, and thereby improve compressive strength.

The above studies indicate that the effects of the type of anti-dispersant, its dosage, and the activator on the properties of alkali-activated slag slurries exhibit significant duality and complexity, and the synergistic regulation mechanism among these three factors remains unclear. In particular, it is unclear how the PAM-HPMC blend system achieves a balance between anti-dispersion and flowability through polymer network regulation during the plastic stage, and how it refines harmful capillaries, optimizes pore size distribution, and thereby enhances compressive strength. Currently, there is a lack of systematic research addressing these issues, making it difficult to provide a scientific basis for mix design.

This study used GGBS as the raw material, liquid sodium silicate with a modulus adjusted by NaOH as the alkali activator, and HPMC and PAM as anti-dispersants. It systematically investigated the effects of the type and dosage of anti-dispersants, as well as the modulus of the activator, on the anti-dispersion properties, rheological behavior, and mechanical properties of alkali-activated slag slurry. Through tests on anti-dispersion, flowability, rheology, and compressive strength, combined with XRD, FT-IR, SEM, energy dispersive spectroscopy (EDS), and MIP analyses, this study elucidates the mechanisms by which PAM and HPMC influence the underwater anti-dispersion and rheological properties of alkali-activated slag, thereby providing a theoretical basis for the mix design of alkali-activated underwater grouting materials.

## 2. Materials and Methods

### 2.1. Materials

#### 2.1.1. GGBS

The precursor material used to prepare alkali-activated slag specimens was S95-grade granulated blast furnace slag powder (GGBS, supplied by Xuzhou Yinshan Rail Concrete Co., Ltd., Xuzhou, China), whose chemical composition is shown in Table 1. In accordance with GB/T 18046-2017 [22] and based on the chemical composition of this GGBS, the alkalinity coefficient $K_{b}=\frac{CaO+MgO}{{SiO}_{2}+{Al}_{2}O_{3}}$ was calculated to be 0.88, and the mass coefficient $Q_{c}=\frac{CaO+MgO+{Al}_{2}O_{3}}{{SiO}_{2}}$ was calculated to be 1.96. The low alkalinity coefficient reflects the acid nature of the slag, which relies on alkaline activators to trigger its hydration reactivity. The high mass coefficient demonstrates abundant reactive oxide components within the slag glass phase, promising excellent latent hydraulic activity. The specific surface area of this slag powder is 416 m^2^/kg, and its density is 2900 kg/m^3^. The particle size distribution was determined using a laser particle size analyzer (Malvern Mastersizer 3000, Malvern Panalytical Ltd., Malvern, UK). The results indicate that the particle size range is 0.1–73 μm, with a median particle size of 12.76 μm.

#### 2.1.2. Alkali Activator

The industrial liquid sodium silicate used in this experiment (Shandong Shengwang Chemical Co., Ltd., Linyi, China) had a moisture content of 63.0 wt%, a Baumé degree of 37°, and a modulus of 3.2. Flake NaOH (Wuxi Zhanwang Chemical Reagent Co., Ltd., Wuxi, China) with a purity of 99.5% was used as the alkaline adjusting agent. The activator solution was prepared by mixing NaOH and liquid sodium silicate according to the designed ratio; the modulus (M = SiO_2_/Na_2_O) of the activator was adjusted by varying the NaOH content. The prepared solution was allowed to cool naturally to room temperature and then sealed for storage until use.

#### 2.1.3. Anti-Dispersant

**Figure 1:**
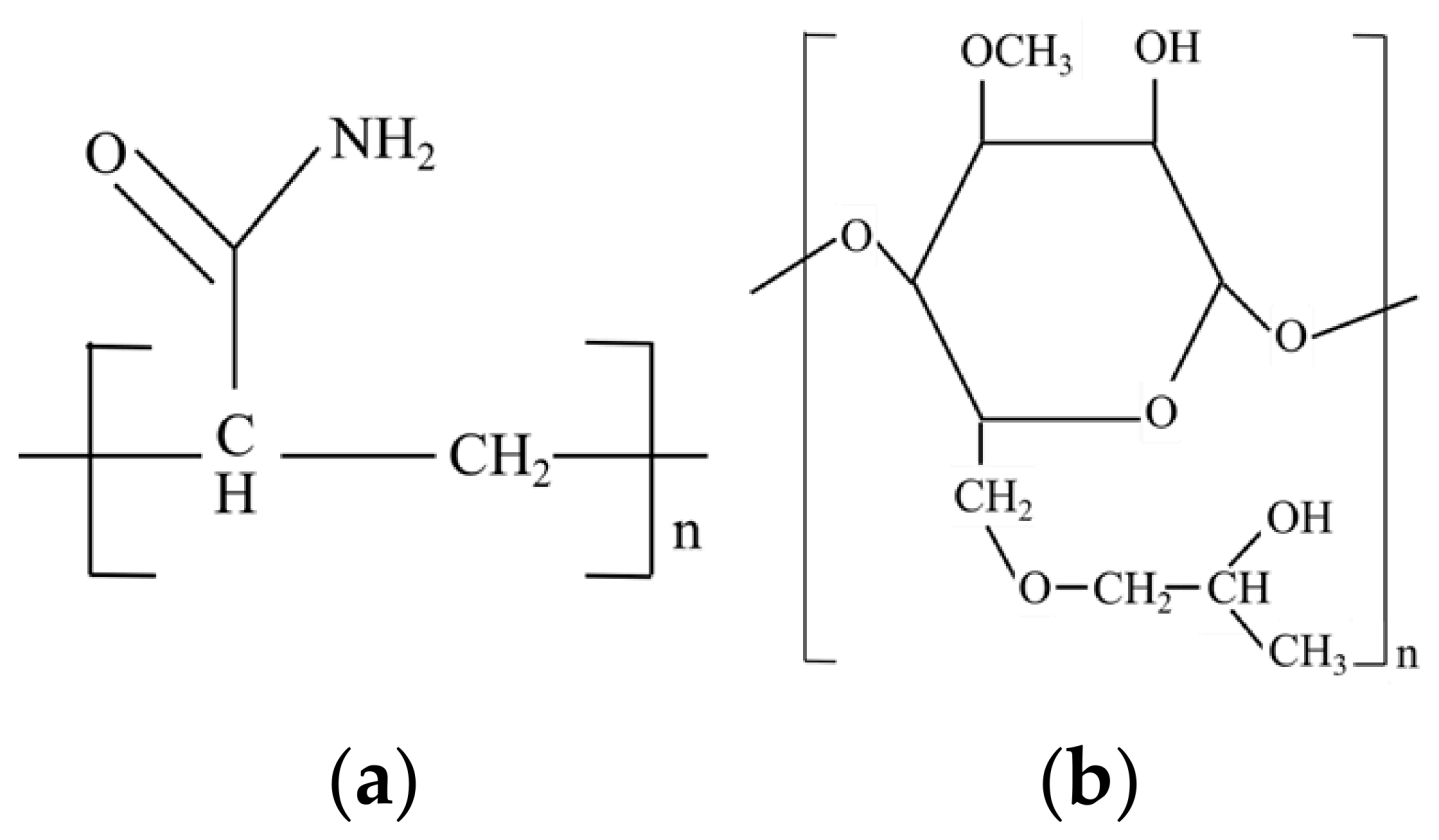
Molecular structures of HPMC and PAM. (**a**) Molecular structure of PAM; (**b**) molecular structure of HPMC.

In this study, hydroxypropyl methylcellulose (HPMC) with a viscosity of 200,000 mPa·s and anionic polyacrylamide (PAM) with a molecular weight of 12 million Da (12 × 10^6^ Da) were selected as anti-dispersants; both were manufactured by Tianjin Huasheng Chemical Reagents Co., Ltd., Tianjin, China. Their chemical structures are shown in Figure 1.

#### 2.1.4. Others

The fine aggregate used was quartz sand (Xiamen ISO Standard Sand Co., Ltd., Xiamen, China), with an apparent density of 2.65 g/cm^3^, a bulk density of 1.91 g/cm^3^, a fineness modulus of 2.54, and a clay content of less than 1.0%. Tap water was used for the tests.

### 2.2. Mix Proportion and Specimen Preparation

#### 2.2.1. Mix Proportion and Specimen Grouping

An L_9_(3^3^) orthogonal array was used to investigate the effects of anti-dispersant type (PAM alone, HPMC alone, and PAM + HPMC blend at 1:1 mass ratio), anti-dispersant dosage (1.0%, 1.5%, and 2.0% by mass of slag), and activator (M = 0.5, 1.0, and 1.5) on the performance of alkali-activated slag slurry. The detailed mix proportions are listed in Table 2. Taking A1 as an example, this denotes an alkali-activated slag slurry containing a single additive of 1.0% PAM and an activator modulus of 0.5. In all tests, the water-to-slag ratio was 0.45, and the liquid sodium silicate content was 15% of the slag mass.

Two additional control groups were included: A0, a blank control without anti-dispersant at M = 1.0, and A10, a test group with 1.0% PAM + HPMC blend (1:1) at M = 1.5. All specimens were prepared and cured under identical conditions. The detailed mix proportions are presented in Table 2.

#### 2.2.2. Sample Preparation

Dry-mix the anti-dispersant with the slag in a mixer(Hebei Xinmingsheng Instrument Equipment Co., Ltd., Cangzhou, China) for 3 min until uniform, then transfer the mixture to a mixing tank(Hebei Xinmingsheng Instrument Equipment Co., Ltd., Cangzhou, China). First, add the alkali activator solution and mixing water, then mix at low speed for 120 s. Stop for 15 s to scrape the slurry from the walls of the mixing tank and the blades, then mix at high speed for another 120 s. Conduct all performance tests immediately after mixing.

To determine the underwater strength of alkali-activated slag slurry, a 40 × 40 × 160 mm^3^ mold was pre-immersed in water prior to pouring the slurry, with the pouring surface maintained 5 cm above the water level. The specimens were demoulded 24 h after casting, and the specimens were then placed in water for curing until the specified age. To simulate real underwater casting conditions and eliminate the influence of manual vibration on the slurry structure, no compaction was applied during casting during the casting process in this study.

### 2.3. Methods

#### 2.3.1. Anti-Dispersion

**Figure 2:**
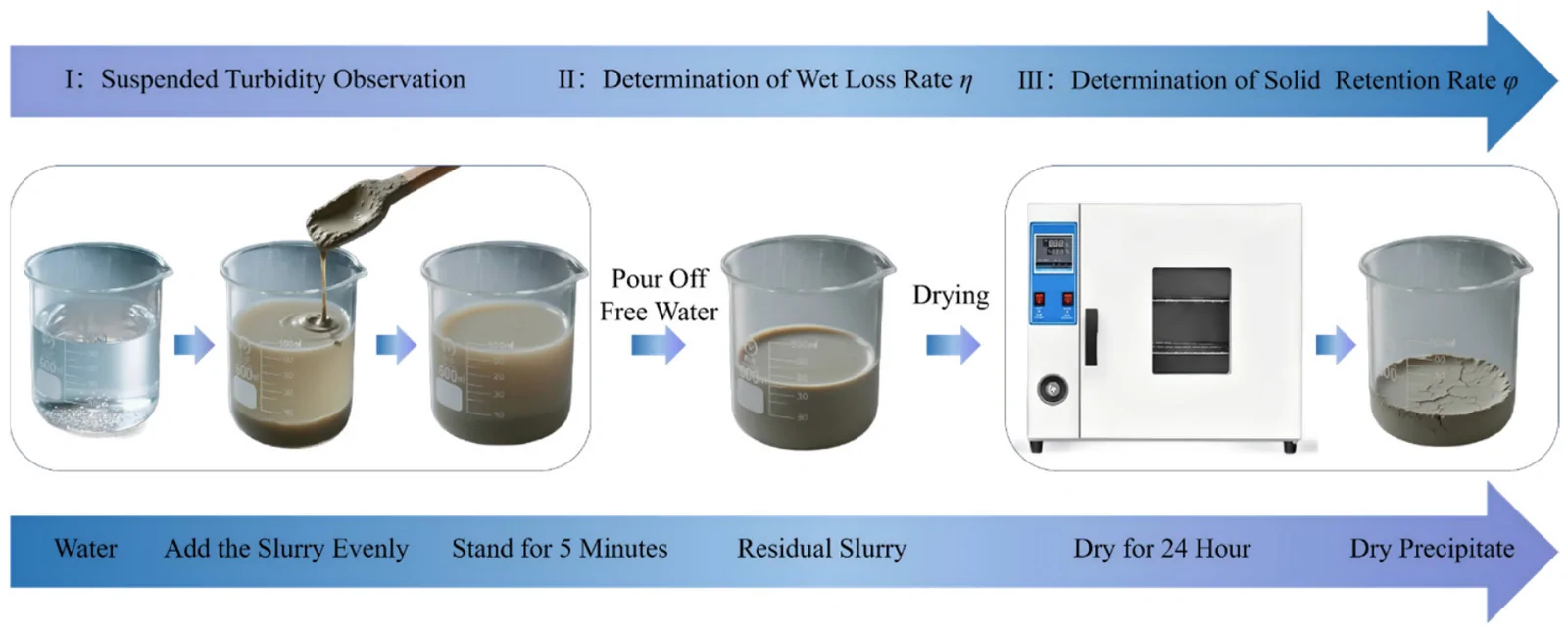
Procedure for determining turbidity, wet loss rate, and solid retention rate of alkali-activated slag slurry.

The test procedure for anti-dispersion is shown in Figure 2.

(1) Suspended turbidity monitoring

Weigh a fresh slurry sample m_0_ (approximately 480 g) and dispense it at a constant rate from a height of 10 cm above the water surface into a 500 mL beaker containing 270 g of distilled water. The total addition time was controlled at 10 s ± 1 s, and the mixture was allowed to stand for 5 min after addition. High-definition video equipment was used to record the process of the slurry falling and its state after settling and stratification; image analysis was then employed to characterize changes in the turbidity of the aqueous phase. Repeat this experiment three times and select images for analysis.

(2) Determination of the wet loss rate of grout during underwater placement

The wet loss rate is an important indicator for evaluating the resistance of fresh mortar to water erosion and its internal cohesion. After allowing the fresh mortar to stand in a beaker for 30 min, decant the free water that has separated from the top of the mortar and the thin slurry floating on the surface, then weigh the mass of the remaining mortar, *m*_1_. The moisture loss rate, *η*, is calculated using Equation (1), where m_0_ is the mass of the freshly mixed slurry (g) and *m*_1_ is the mass of the residual slurry (g). Each set of tests was repeated three times, and the average was calculated.

(1) $$\;\eta =\frac{m_{0}+270-m_{1}}{m_{0}+270}\times 100\%$$

(3) Determination of solid-phase retention in underwater-poured grout

The solid-phase retention rate is used to characterize a grout’s ability to retain cementitious materials. After completing the test for the wet loss rate of the grout poured underwater, place the beaker containing the residual grout in an oven at 110 ± 5 °C and dry it for 24 h. After cooling to room temperature (20 ± 2 °C), weigh the mass *m*_2_ of the dried precipitate, and calculate the solid-phase retention rate *φ* according to Equation (2). In the equation, *m*_2_ represents the mass of the dried precipitate (g). Each set of tests was repeated three times, and the average was calculated.

(2) $$\varphi =\frac{m_{2}}{m_{1}}\times 100\%$$

It should be noted that this indicator directly reflects the slurry’s ability to physically bind solid particles. Drying at 110 °C causes some bound water to be lost and results in a small amount of polymer pyrolysis, leading to lower absolute values for solid-phase retention in the test results. However, since all samples underwent the same drying and testing procedures, systematic errors arising from testing conditions were eliminated; therefore, this study uses the relative differences between groups as the primary evaluation criterion.

#### 2.3.2. Rheology

(1) Fluidity

The flowability of alkali-activated slag slurry was measured via a truncated cone mold test in accordance with GB/T 8077-2023 [23]. A standard truncated cone mold (top inner diameter of 36 mm, bottom inner diameter of 60 mm, and height of 60 mm) was placed centrally on a glass plate. After the freshly prepared slurry was filled into the mold, the mold was vertically lifted, and the slurry was allowed to flow freely for 30 s. The spreading diameters in two orthogonal directions were subsequently measured and averaged. Each mixture was tested in triplicate, and the mean value of the three replicates was adopted as the final flowability result.

(2) Rheological model

Geopolymer slurries have been shown to be non-Newtonian fluids, and their rheological behavior is typically described using the Bingham, Herschel–Bulkley (HB), and Modified Bingham (MB) models [24,25]. Numerous studies have shown that alkali-activated slag slurries exhibit significant nonlinear rheological characteristics under low water-to-slag ratio conditions, and the Bingham model struggles to accurately describe their rheological behavior [24]. Although the HB model introduces a consistency coefficient and a rheological index to characterize slurry flow, it differs significantly from the traditional characterization system based on yield stress and plastic viscosity [26]; the MB model, by introducing a quadratic term coefficient, is able to better describe the nonlinear shear behavior of the slurry while retaining yield stress and plastic viscosity [26]. Therefore, this study selected the MB model to fit the rheological behavior of alkali-activated slag slurry, and its mathematical expression is shown in Equation (3).

(3) $$\tau =\tau_{0}+\eta \dot{\gamma }+c{\dot{\gamma }}^{2}$$

In this equation, *τ* represents shear stress (Pa), *τ*_0_ represents yield stress (Pa), *η* is the plastic viscosity (Pa·s), $\dot{\gamma }$ represents shear rate (s^−1^), and *c* is the consistency coefficient, which is used to describe the shear-thinning or shear-thickening behavior of the slurry.

(3) Rheological Testing

Rheological testing was conducted using a Brookfield RST 3000 rotational rheometer (AMETEK Brookfield, Middleboro, MA, USA), following the test procedure outlined in ASTM C1749-25 [27], “Standard Guide for Measurement of the Rheological Properties of Hydraulic Cementitious Paste Using a Rotational Rheometer.” A V80-40 paddle rotor with a paddle height of 40 mm and a diameter of 20 mm was used. All measurements were performed at a controlled temperature of 20 ± 1 °C. Prior to testing, to standardize the initial state of the slurry, it was pre-sheared for 60 s at a constant shear rate of 60 s^−1^, followed by a 30-s rest period to eliminate residual stress. During the formal test, the shear rate was linearly increased from 0 to 100 s^−1^ over 60 s, and then decreased from 100 s^−1^ to 0 s^−1^ over the same time period. To eliminate disturbances caused by the pre-shearing, the parameters fitted from the deceleration phase were adopted as the rheological parameters representing the slurry’s equilibrium state. To ensure the reproducibility of the results, three sets of rheological tests were conducted for each mix design.

#### 2.3.3. Initial Setting Time

The initial setting time refers to the duration from mixing until the paste loses plasticity. Vicat apparatus (Zhejiang Guangnian Zhixin Instrument Co., Ltd., Shaoxing, China) was used to test the initial setting time of alkali-activated slag pastes following GB/T 1346–2024 [28]. Three parallel samples were tested per group, and averaged data were recorded.

#### 2.3.4. Compressive Strength

The alkali-activated slag mortar test specimens measured 40 mm × 40 mm × 160 mm and were prepared in accordance with the “Test method of cement mortar strength (ISO method )” (GB/T 17671-2021) [29]. Three specimens were tested for each mix design at each curing age, and the average compressive strength was calculated.

#### 2.3.5. XRD Analysis

The crystalline phase composition of samples was characterized via X-ray diffraction (XRD; Rigaku Ultima IV, Rigaku Corporation, Akishima, Japan). The test parameters were set as an accelerating voltage of 40 kV, a tube current of 40 mA, a scanning range of 2θ = 10–70°, and a scanning rate of 2°/min.

#### 2.3.6. FT-IR Analysis

Fourier-transform infrared spectroscopy (FTIR; VERTEX 80v, Bruker Optik GmbH, Ettlingen, Germany) was employed to characterize molecular vibrational features of the samples. The spectra were recorded in the wavenumber range of 500–4000 cm^−1^ with a spectral resolution of 2 cm^−1^ and 32 accumulated scans. OPUS 7.5 software (Bruker Optik GmbH, Ettlingen, Germany) was utilized for the identification and analysis of characteristic absorption peaks.

#### 2.3.7. SEM and EDS

A scanning electron microscope (SEM; GAIA3 XMH, TESCAN GROUP, a.s., Brno, Czech Republic) coupled with an energy dispersive spectrometer (EDS; GAIA3 XMH, TESCAN GROUP, a.s., Brno, Czech Republic) was used to examine the microstructure and perform elemental surface scanning analysis of the alkali-activated slag slurry after 7 days of hydration, with an acceleration voltage of 10 kV and a working distance of 5.2 mm [30]. The hydration reaction was terminated using the solvent-exchange method: the samples were immersed in anhydrous ethanol for 72 h, with the solvent replaced three times during this period, and subsequently dried in an oven at 50 ± 1 °C for 24 h. The samples were gold-sputtered prior to observation.

#### 2.3.8. Porosity

**Figure 3:**
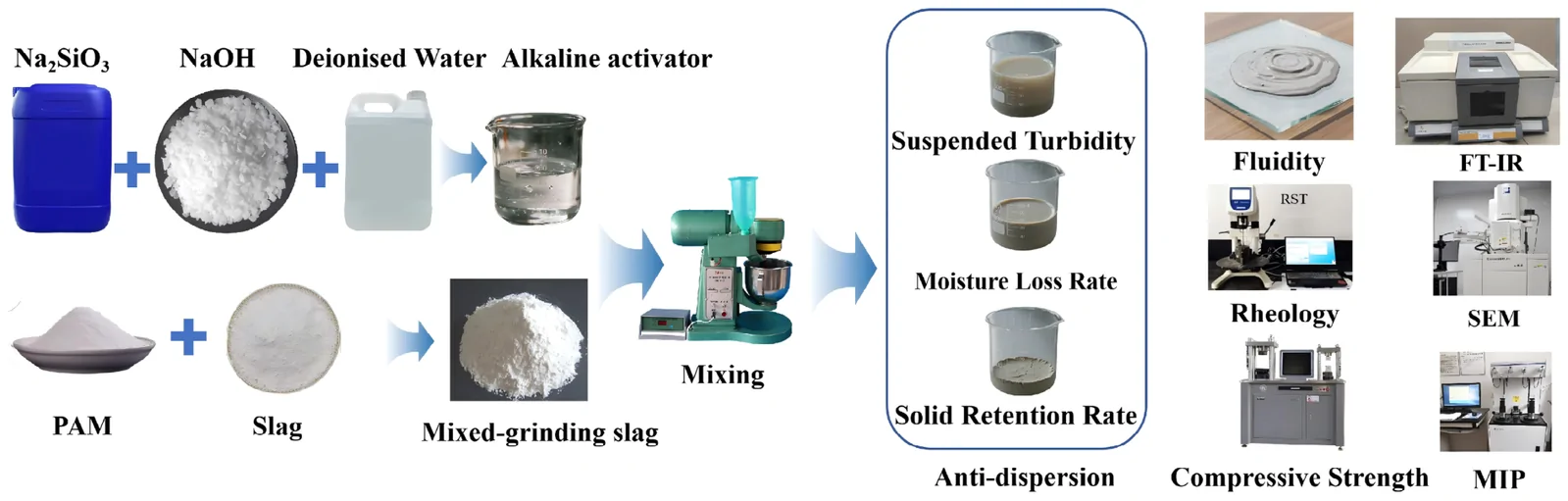
Experimental flowchart.

The pore structure of the hardened slurry was tested using a PoreMaster 60 fully automatic mercury porosimeter (Quantachrome Instruments, Boynton Beach, FL, USA), with a maximum pressure of 414 MPa and an effective pore size measurement range of 3.6 nm to 1100 µm. Hydration was terminated using the same solvent displacement method as in the SEM analysis. The overall technical approach of this study is shown in Figure 3.

#### 2.3.9. Range Analysis

Range analysis evaluates the degree of influence of each factor by calculating the statistical parameters of performance indicators at different levels of the factor. The K-value (the average of the performance metrics for a given factor at a single level) is used to determine the optimal level for each factor; the R-value (range) is used to preliminarily measure the extent of each factor’s impact on performance; and the *p*-value (the significance probability from analysis of variance) is used to determine whether each factor has a significant effect on the corresponding performance metric. Finally, by comprehensively comparing the K-values, R-values, and *p*-values of each factor, the optimal combination that balances multiple performance metrics is determined.

## 3. Results and Discussion

### 3.1. Effect of Antidispersants and Activator on the Dispersion Resistance of Alkali-Activated Slag Slurry

#### 3.1.1. Observation of Turbidity in Alkali-Activated Slag Slurry

**Figure 4:**
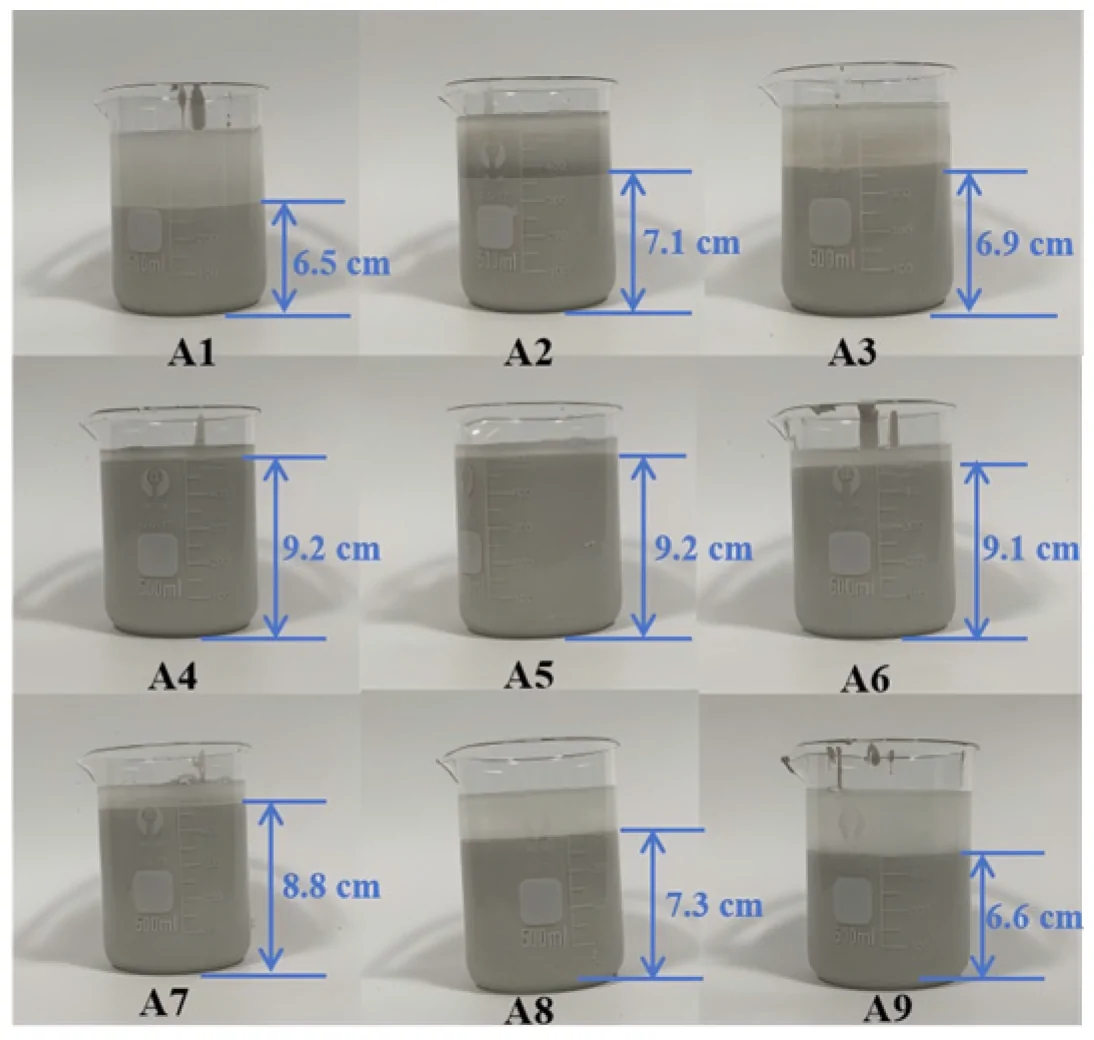
Stratification and sedimentation height of alkali-activated slag slurry.

Figure 4 shows photographs of the turbidity of alkali-activated slag slurry after standing for 5 min under different types of anti-dispersants, dosage levels, and activator modulus. As shown in Figure 4, there are significant differences in the stratification characteristics and the state of free water in the upper layer among the various systems.

When PAM was added alone (A1, A2, A3), the slurry exhibited distinct layering and a significant flocculation effect; however, the upper layer of free water remained turbid throughout, indicating that PAM has limited ability to trap and settle fine particles, with some microfine particles still suspended in the aqueous phase. The sedimentation heights for the three groups were 6.5 cm, 7.1 cm, and 6.9 cm, respectively, with the lowest sedimentation height observed in the slurry containing 1% PAM with a modulus of 0.5.

When HPMC was added alone (A4, A5, A6), the upper layer of free water decreased sharply, and there was no distinct interface between the slurry layer and the aqueous phase; a large number of particles were dispersed in the aqueous phase, and the turbidity of the slurry increased significantly. The sedimentation heights for the three groups were 9.2 cm, 9.2 cm, and 9.1 cm, respectively, indicating that HPMC has excellent water retention but insufficient flocculation capabilities.

After co-blending PAM and HPMC (A7, A8, A9), the free water in the upper layer was relatively clear, with sedimentation heights of 8.8 cm, 7.3 cm, and 6.6 cm, respectively, and a distinct interface between the slurry and water.

PAM forms a flocculation network through bridging effects [13], while HPMC binds free water via hydrogen bonding and enhances the cohesive force within the slurry [11]; the synergistic action of these two agents optimizes particle aggregation and effectively suppresses the suspension of fine particles. Based on a comprehensive evaluation of both sedimentation height and the clarity of the free water, the co-blended system outperformed the single-additive systems in terms of both solid–liquid separation efficiency.

#### 3.1.2. Effect of Antidispersant and Activator on the Wet Loss Rate of Alkali-Activated Slag Slurry

(1) Analysis of moisture loss rates

**Figure 5:**
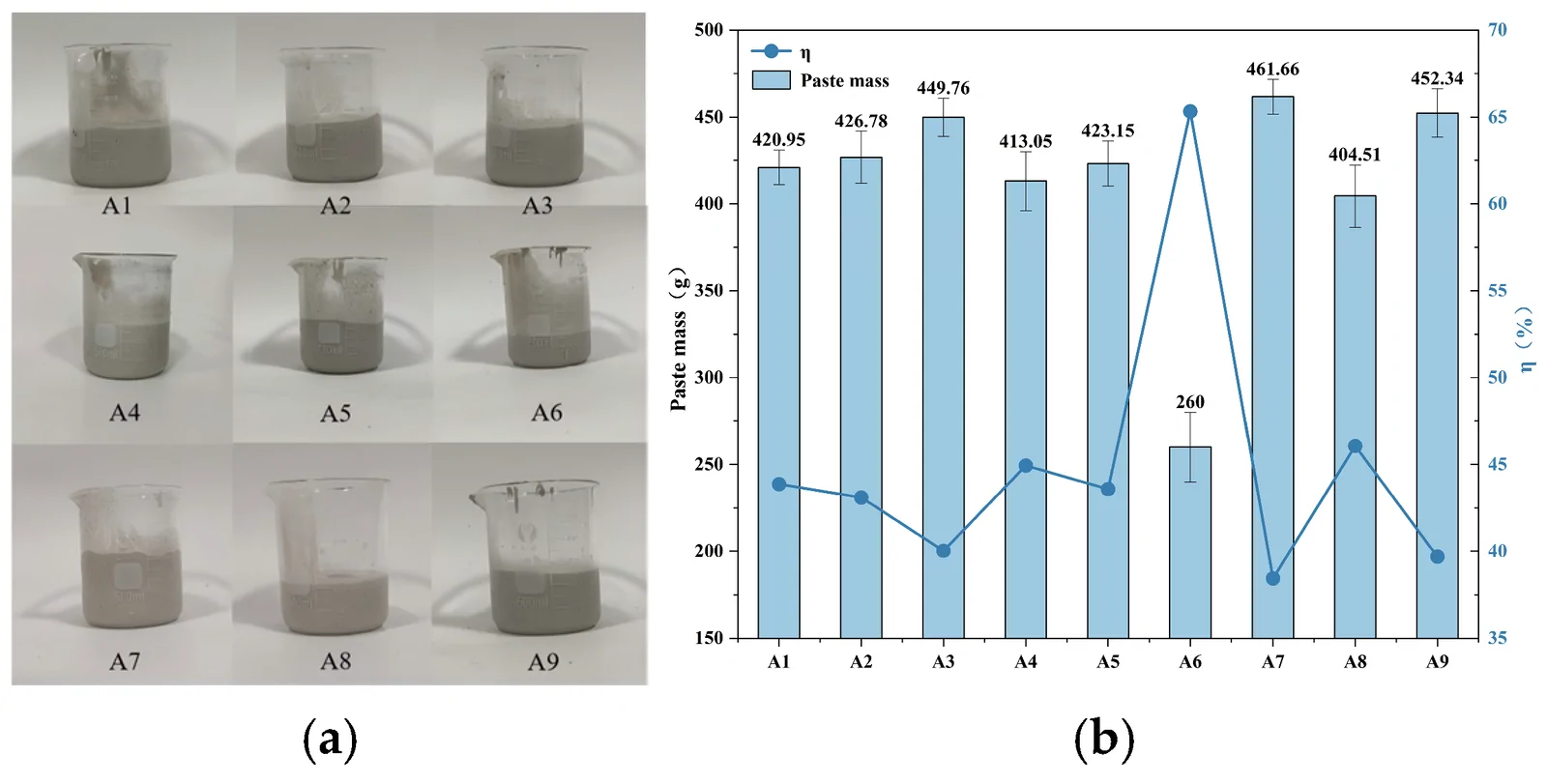
Alkali-activated slag pastes and their mass loss rates. (**a**) Alkali-activated slag slurry with free water and thin slurry removed; (**b**) Alkali-activated slag slurry wet loss rate *η*.

The wet loss rate is an important indicator for evaluating the resistance of fresh slurry to water erosion and its cohesion properties; the lower the value, the stronger the slurry’s ability to resist erosion and particle loss. The residual state of alkali-activated slag slurry after removing free water and dilute slurry is displayed in Figure 5a, which reflects the water erosion resistance of different slurry samples.

As shown in Figure 5b, the wet loss rates for each group ranged from 38.45% to 65.33%, and the residual slurry mass ranged from 260.00 g to 461.66 g. Among them, A6 (modulus 0.5, 2% HPMC single admixture) had the highest wet loss rate (65.33%) and the lowest slurry mass (260.00 g). This may be due to the combined effect of the low-modulus activator and the high HPMC content, which weakened the uniformity of the slurry structure, leading to the massive release of free water that diluted the internal structure of the slurry and reduced its scour resistance.

At M = 1.0, composite system A7 exhibited the lowest wet loss rate (38.45%) and the highest residual mass (461.66 g), significantly outperforming the PAM-only system A3 (40.03%, 449.76 g) and the single-additive HPMC system A5 (43.58%, 423.15 g) at the same modulus. Furthermore, A7 had the lowest total dispersant dosage (1.0%), indicating that the blended system is more efficient than the single-additive systems. Further comparison of A7 (M = 1.0), A8 (M = 0.5, 46.07%), and A9 (M = 1.5, 39.69%)—all of which are blended systems—shows that A7 has the lowest wet loss rate and the smallest dosage, indicating that a modulus of 1.0 is optimal. This may be because, at this modulus, the alkaline activator fully depolymerizes the slag; the leached active ions condense with the silicate groups in the liquid phase to form a continuous, uniform hydrated gel matrix. At the same time, PAM and HPMC synergistically reinforce the skeletal structure, enhancing interparticle cohesion and solid-phase retention capacity, thereby imparting optimal scour resistance to the slurry.

(2) Range analysis of slurry wet loss rate

**Figure 6:**
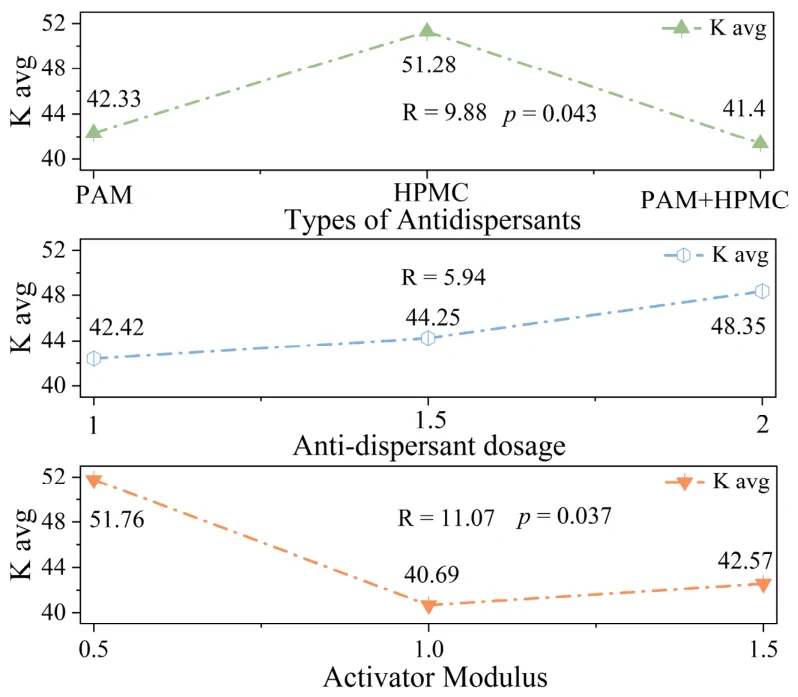
Range analysis of the wet loss rate of alkali-activated slag slurry.

The results of the range analysis for the moisture loss rate are shown in Figure 6. The degree of influence of each factor, from greatest to least, is as follows: activator modulus (R = 11.07) > type of anti-dispersant (R = 9.88) > anti-dispersant dosage (R = 5.94). An analysis of variance was conducted using the anti-dispersant dosage with the smallest range as a dummy error term. The results showed that the *p*-values for both the activator modulus (*p* = 0.037) and the type of anti-dispersant (*p* = 0.043) were less than 0.05, indicating statistical significance. Based on the analysis of variance, the confidence interval for the optimal combination is (36%, 43.42%). Combining the optimal levels of all factors, the alkali-activated slag slurry exhibits the best resistance to water scouring when the activator modulus is 1.0, the anti-dispersant dosage is 1%, and a blend of PAM and HPMC is used.

#### 3.1.3. Effect of Antidispersant and Activator on the Solid-Phase Retention Rate of Alkali-Activated Slag Slurry

(1) Analysis of solid-phase retention

**Figure 7:**
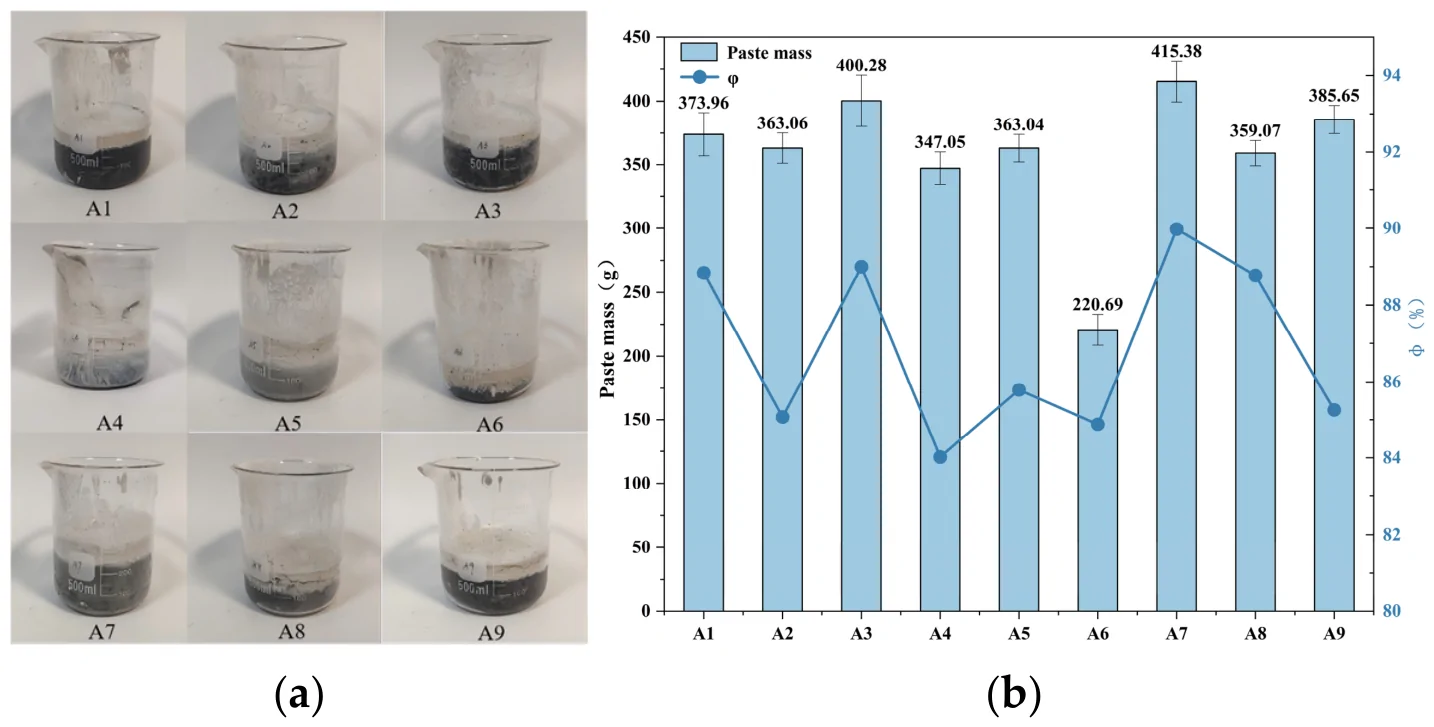
Mass of alkali-activated slag slurry after 24 h of drying and its solid-phase retention rate *φ* (%). (**a**) Alkali-activated slag slurry after 24 h of drying; (**b**) Alkali-activated slag slurry quality and solid-phase retention rate *φ* (%).

Solid-phase retention is a key indicator for evaluating a slurry’s ability to bind slag particles; a higher value indicates a greater content of bound water within the slurry, a lower proportion of free water, and superior cohesion and anti-dispersion properties. Figure 7a presents the morphological characteristics of the dried slurry residues, providing visual support for the analysis of solid-phase retention performance.

As shown in Figure 7b, Groups A4–A6 (single-doped with HPMC) exhibited lower solid-phase retention rates (84.02–85.79%), primarily due to the strong water-retention properties of HPMC. During the drying process, HPMC gradually released the large amount of free water it had adsorbed; as the water evaporated, the number of capillaries within the slurry increased and its structure became more porous, leading to a significant decrease in the solid-phase retention rate. A1, A3, A7, and A8 exhibited higher and similar solid-phase retention rates (88.77–89.98%), indicating that when the modulus of the activator is below 1.5, both the single-blended PAM system and the composite system of PAM and HPMC can effectively enhance the slurry’s ability to bind slag particles and retain bound water.

HPMC achieves water retention and thickening primarily by forming hydrogen bonds with water molecules through its hydroxyl and ether groups, whereas it lacks effective particle binding capacity and cannot firmly constrain solid particles. In contrast, PAM, rich in amide groups, can tightly adsorb onto the surface of slag particles. Its long molecular chains further bridge and connect dispersed particles to construct a continuous and stable flocculation network, which effectively restricts particle migration and reduces solid loss.

(2) Range analysis of slurry solid-phase retention rate

**Figure 8:**
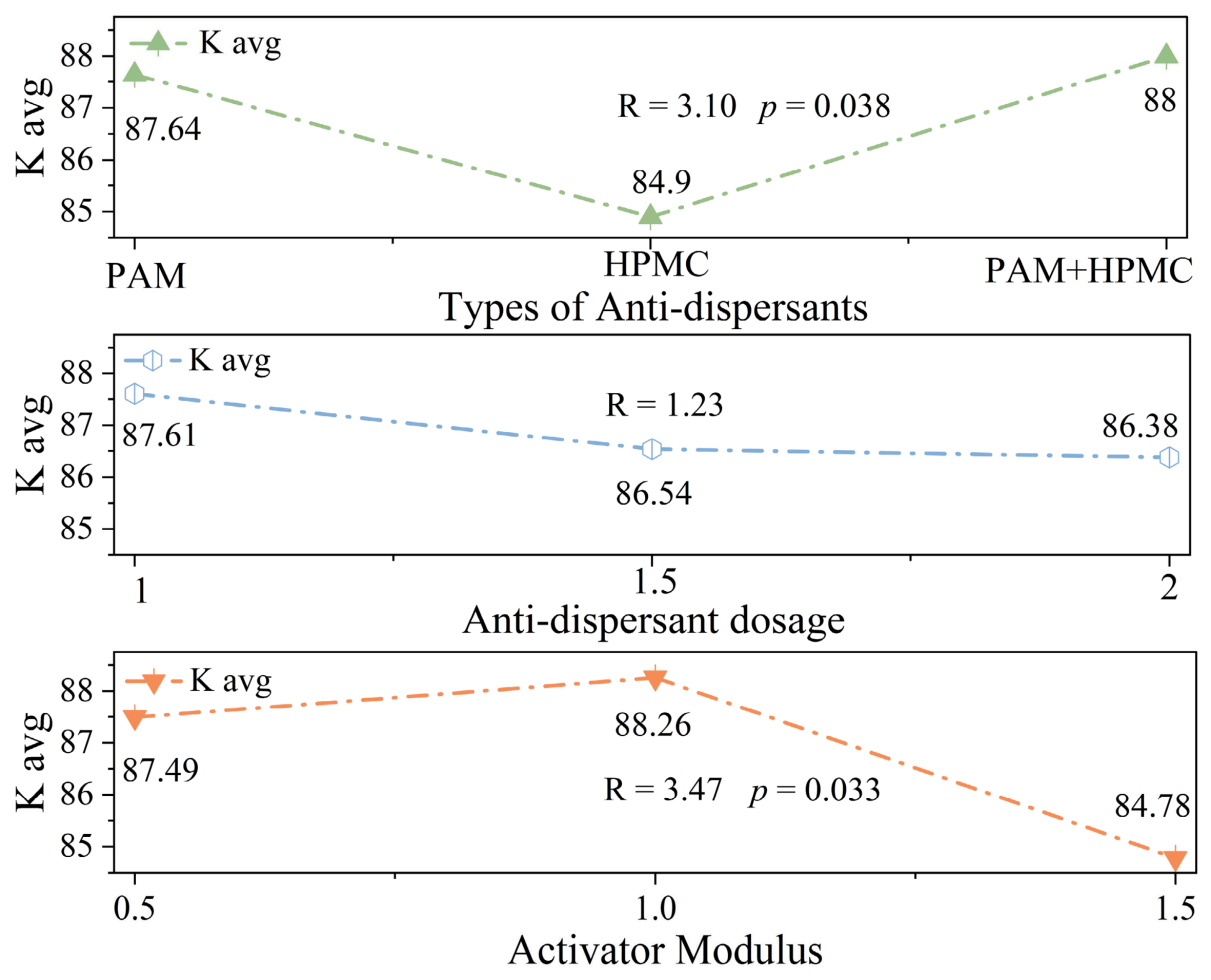
Range analysis of solid-phase retention in alkali-activated slag slurry.

The results of the range analysis for solid-phase retention are shown in Figure 8. The degree of influence of each factor, from greatest to least, is as follows: activator modulus (R = 3.47) > type of anti-dispersant (R = 3.10) > anti-dispersant dosage (R = 1.23). The range values for the activator modulus and the type of anti-dispersant were relatively close, indicating that both are important factors affecting the slurry’s particle binding capacity. An analysis of variance was conducted using the anti-dispersant dosage with the smallest range as a dummy error term. The results showed that the *p*-values for both the activator modulus (*p* = 0.033) and the type of anti-dispersant (*p* = 0.038) were less than 0.05, reaching statistical significance. Based on the analysis of variance, the confidence interval for the optimal combination is (88.52%, 91.44%). Considering the optimal levels of all factors, the alkali-activated slag slurry exhibits the best particle binding capacity when the activator modulus is 1.0, the anti-dispersant dosage is 1%, and a blend of PAM and HPMC is used.

### 3.2. Effect of Anti-Dispersant and Activator on the Flowability and Rheological Properties of Alkali-Activated Slag Slurry

#### 3.2.1. Effect of Antidispersant and Activator on the Flowability of Alkali-Activated Slag Slurry

(1) Flowability analysis

**Figure 9:**
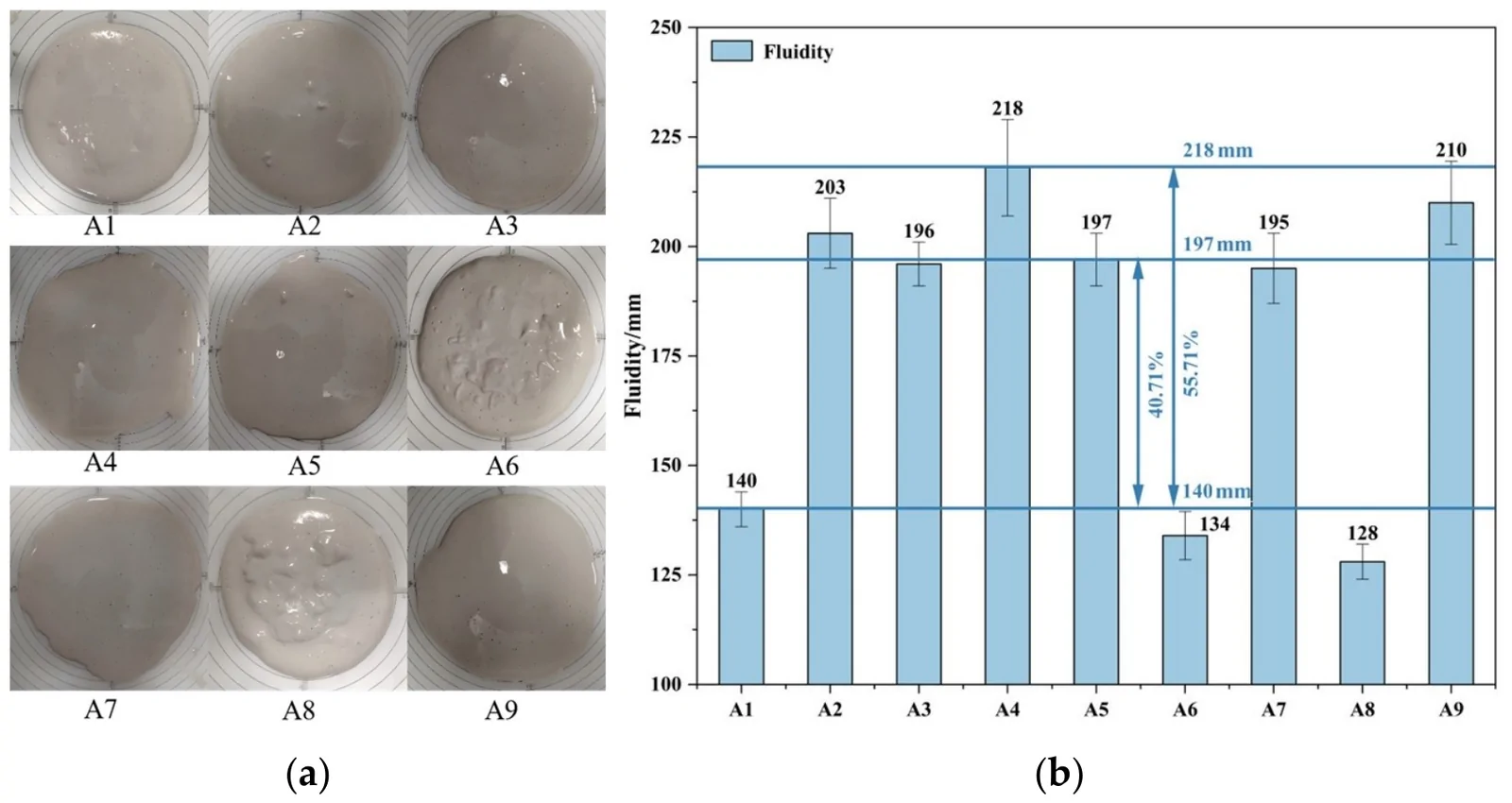
Effect of antidispersant and activator modulus on the flowability of alkali-activated slag slurry. (**a**) Photo of the flowability of alkali-activated slag slurry; (**b**) alkali-activated slag slurry flowability.

The macroscopic flow behavior of different alkali-activated slag slurries during the flowability test is presented in Figure 9a. Corresponding quantitative results are summarized in Figure 9b, where the flowability of each group falls primarily into three distinct ranges, corresponding to sodium silicate moduli of 0.5 (A1, A6, and A8), 1.0 (A3, A5, and A7), and 1.5 (A2, A4, and A9), with values of 128–140 mm, 195–197 mm, and 203–218 mm, respectively. As the sodium silicate modulus increases, the slurry fluidity shows a continuous upward trend. Under the same modulus conditions, although there are differences in the type and dosage of anti-dispersants, the degree of dispersion in the fluidity data within each group is relatively small, indicating that the activator is the dominant factor determining fluidity, while the types and dosages of anti-dispersants have a relatively limited effect on fluidity.

Since fluidity reflects a slurry’s ability to spread under its own weight, it depends on the frictional resistance between solid particles and the strength of the floc structure. The lower the activator modulus, the higher the liquid-phase OH^−^ concentration, and the more intense the early-stage depolymerization of the slag glass matrix. Hydration products form rapidly on the particle surfaces and interlock to form a flocculation network, thereby reducing fluidity; The higher the modulus, the lower the liquid-phase alkalinity; early hydration is inhibited, the flocculation structure between particles weakens, and soluble silicate ions adsorb onto the slag particle surfaces, acting as dispersants and lubricants to further reduce interparticle frictional resistance, thereby increasing fluidity. Anti-dispersants primarily form flocculation networks, increasing interparticle cohesion and shear resistance; their function essentially involves regulating flow resistance. Therefore, the flowability of the slurry is primarily governed by the activator.

(2) Range analysis of slurry flowability

**Figure 10:**
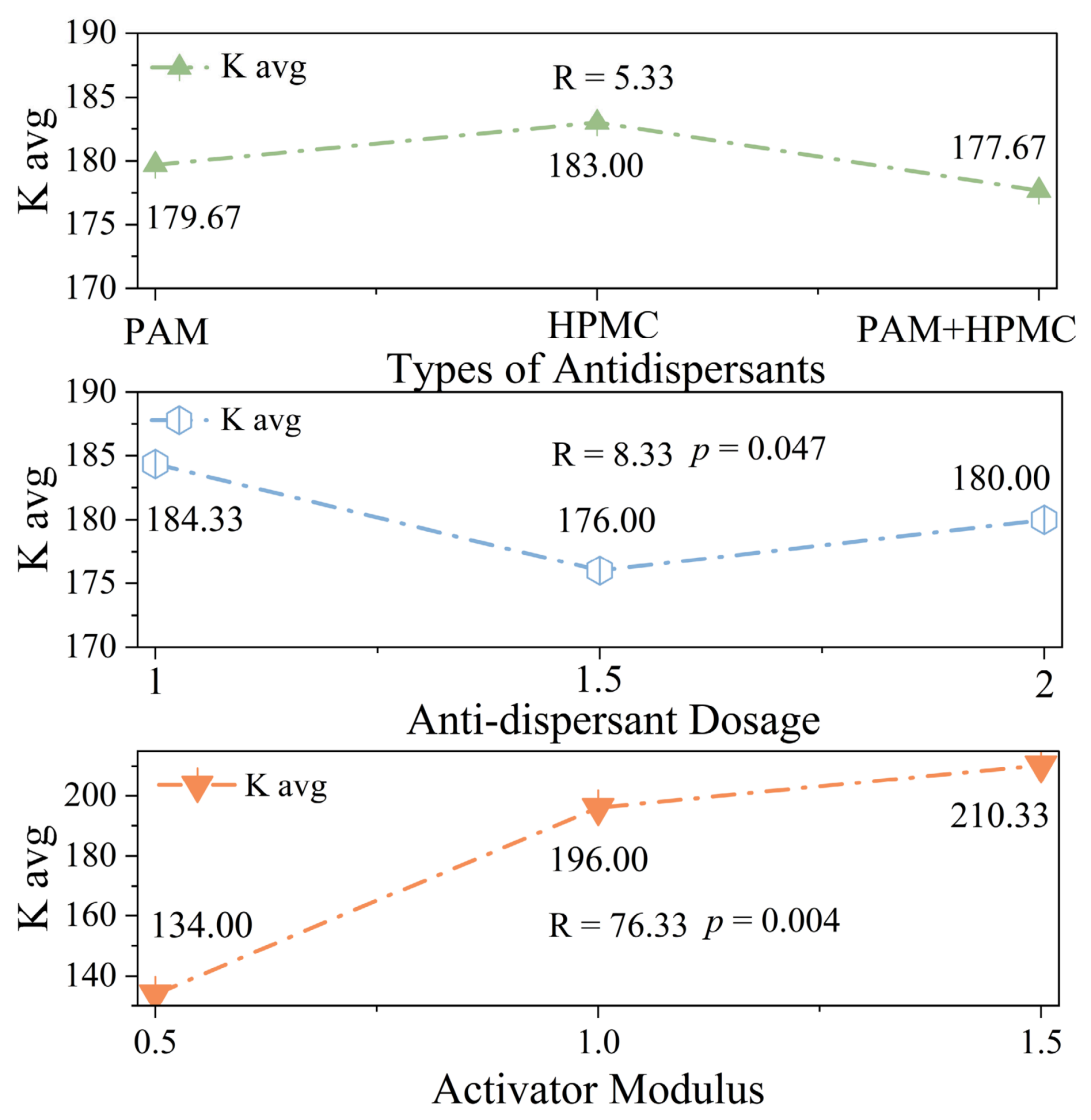
Range analysis of flowability in alkali-activated slag slurry.

The results of the analysis of extreme differences in flowability are shown in Figure 10. The degree of influence of each factor, from greatest to least, is as follows: activator modulus (R = 76.33) > anti-dispersant dosage (R = 8.33) > anti-dispersant type (R = 5.33). An analysis of variance was conducted using the type of anti-dispersant with the smallest range as a dummy error term. The results showed that the *p*-values for both the modulus of the activator (*p* = 0.004) and the anti-dispersant dosage (*p* = 0.047) were less than 0.05, indicating statistical significance. Based on the analysis of variance, the confidence interval corresponding to the optimal combination is (206.94, 229.06) mm. Considering the optimal levels of all factors, the slurry exhibits the best flowability when the activator modulus is 1.5, the anti-dispersant dosage is 1%, and HPMC is used as the sole additive.

#### 3.2.2. Effects of Anti-Dispersant and Activator on the Yield Stress and Plastic Viscosity of Alkali-Activated Slag Slurry

(1) Analysis of MB model fitting for alkali-activated slag slurry

**Figure 11:**
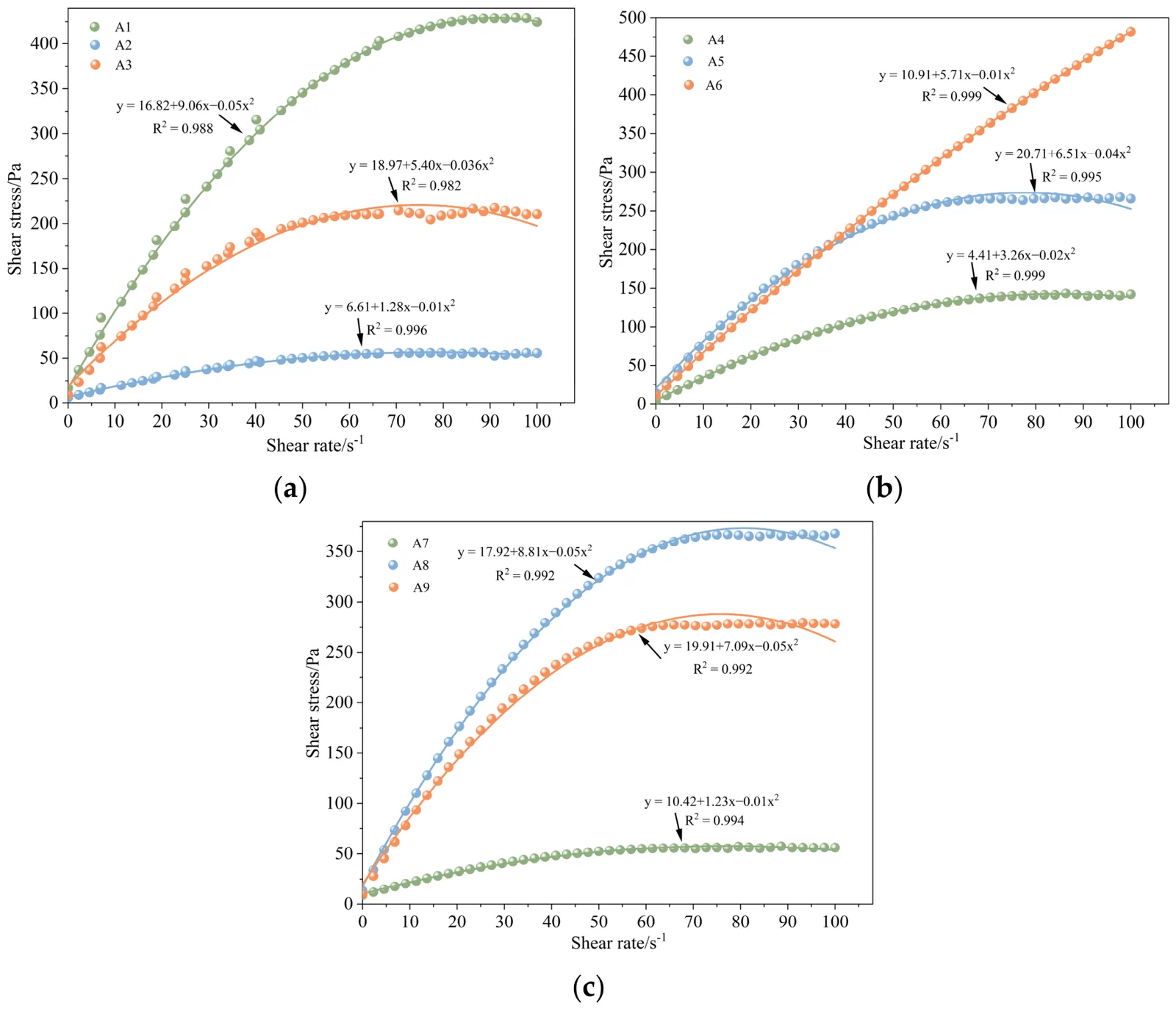
Rheological modeling of alkali-activated slag slurry under different types of antidispersants, dosages, and activator moduli. (**a**) The anti-dispersant is PAM; (**b**) the anti-dispersant is HPMC; (**c**) the anti-dispersants are PAM and HPMC.

The results of MB model fitting for alkali-activated slag slurry under different types of anti-dispersants, dosage levels, and activator moduli are shown in Table 3 and Figure 11. The R^2^ values for all fitting curves exceeded 0.98, indicating that the model can accurately describe their rheological behavior. With regard to yield stress, it is significantly influenced by the activator in single-additive systems.

Taking a single PAM addition as an example, the yield stresses of A1 (modulus 0.5), A2 (modulus 1.5), and A3 (modulus 1.0) have yield stresses of 16.82 Pa, 6.61 Pa, and 18.97 Pa, respectively. At low modulus levels, the higher alkalinity of the activator accelerates the deagglomeration of slag particles and early hydration, resulting in a dense flocculation structure between particles, which in turn increases the resistance to flow initiation. In the blended system, the yield stress is primarily influenced by the dosage of the anti-dispersant and increases continuously as the dosage increases.

In terms of plastic viscosity, in the single-blend system, the plastic viscosity reached its lowest value across all groups at a modulus of 1.5, indicating that at a modulus of 1.5, interparticle frictional resistance decreases and the system’s dispersibility improves. In a compound blending system, when the total content of anti-dispersants exceeds 1%, the plastic viscosity increases significantly. This is because, as the anti-dispersant content increases, the number of polymer chains per unit volume rises, interchain entanglement intensifies, and intermolecular interactions per unit volume increase sharply [31], thereby increasing internal friction resistance and resistance to flow deformation. Furthermore, the consistency coefficients (c) for A1–A9 were all negative, exhibiting shear-thinning behavior; that is, as the shear rate increased, the flocculation structure within the slurry was gradually disrupted, and the flow resistance decreased.

(2) Range analysis of rheological parameters

**Figure 12:**
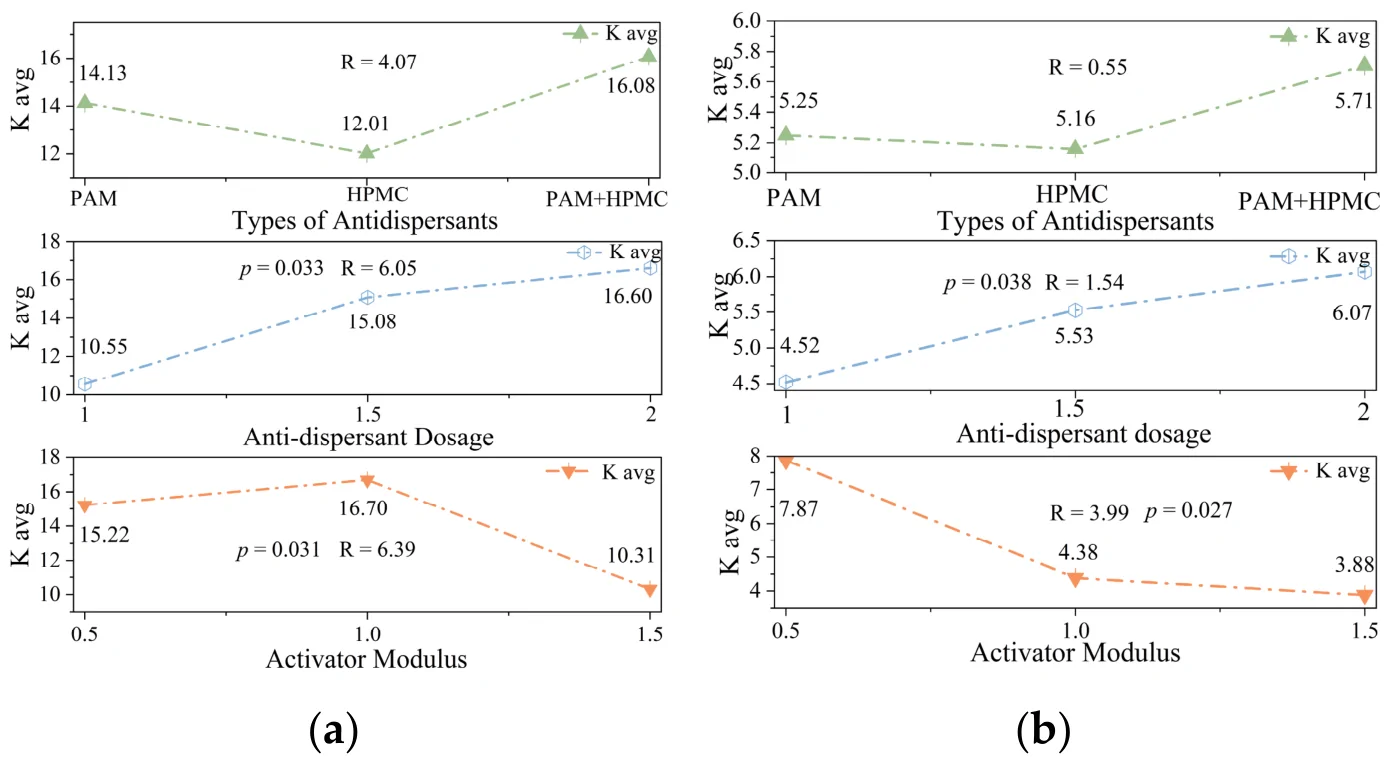
Range analysis of yield stress and plastic viscosity in alkali-activated slag slurry. (**a**) Analysis of yield strengths; (**b**) analysis of plastic viscosity.

The results of the range analysis for yield stress and plastic viscosity are shown in Figure 12. The ranking of the effects of each factor on yield stress is as follows: activator modulus (R = 6.39) > anti-dispersant dosage (R = 6.05) > anti-dispersant type (R = 4.07). For plastic viscosity, the order of influence is: activator modulus (R = 3.99) > anti-dispersant dosage (R = 1.54) > anti-dispersant type (R = 0.55).

An analysis of variance was performed using the anti-dispersant type with the smallest range as a dummy error term. The results showed that for yield stress, the *p*-values for the activator modulus (*p* = 0.031) and the anti-dispersant dosage (*p* = 0.033) were both less than 0.05. For plastic viscosity, the *p*-values for the activator modulus (*p* = 0.027) and the anti-dispersant dosage (*p* = 0.038) were both less than 0.05, indicating statistical significance. Based on the analysis of variance, the confidence intervals corresponding to the optimal combination were (2.13, 6.69) Pa for yield stress and (1.59, 4.93) Pa·s for plastic viscosity. Taking into account the optimal levels of all factors, the yield stress and plastic viscosity both reached their minimum values when the activator modulus was 1.5, the anti-dispersant content was 1%, and HPMC was used as the sole additive.

### 3.3. Effect of Antidispersant and Activator on the Initial Setting Time of Alkali-Activated Slag

(1) Initial setting time analysis

Initial setting time is a key indicator for evaluating the hydration process of the slurry and the construction window; the shorter the setting time, the faster the slurry loses its plasticity and forms a continuous gel network.

**Figure 13:**
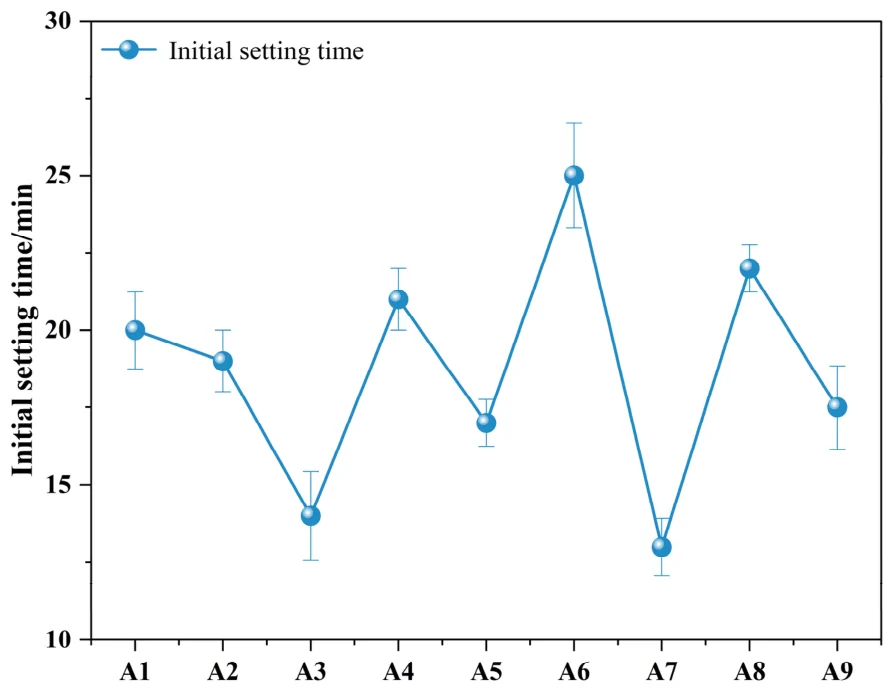
The initial setting time of alkali-activated slag.

As shown in Figure 13, the initial setting times for each group ranged from 13 to 25 min. Among them, A6 (modulus 0.5, 2% HPMC single admixture) had the longest initial setting time of 25 min. This may be due to an insufficient supply of soluble silicate ions in the system at low modulus, making it difficult to continuously consume the leached Ca^2+^ and Al^3+^ ions and preventing the rapid formation of a continuous C-(A)-S-H gel matrix within the slurry. Combined with the retarding effect of HPMC, these factors collectively prolong the initial setting time.

For PAM-single-doped, HPMC-single-doped, and hybrid PAM-HPMC systems, the setting time reaches the minimum value at the activator modulus of 1.0. Among all batches, group A7 (modulus = 1.0, 1 wt% PAM-HPMC hybrid) exhibits the shortest initial setting time of 13 min. This can be attributed to the optimal matching between liquid-phase alkalinity and soluble silicate concentration at this modulus, which synchronizes the depolymerization of slag glassy phase and gel polymerization. Meanwhile, the low dosage of 1 wt% PAM-HPMC imposes the weakest retarding effect on hydration. These two combined factors facilitate the rapid formation of a continuous gel network.

At modulus 0.5, despite the highest alkalinity, insufficient supply of soluble silicate prevents hydration products from evolving into a percolating continuous gel matrix. At modulus 1.5, sufficient silicate is available yet the low alkalinity restricts the overall depolymerization of slag. The optimum coupling of alkalinity and soluble silicate at modulus 1.0 therefore yields the fastest formation of gel matrix.

(2) Range analysis of slurry initial setting time

**Figure 14:**
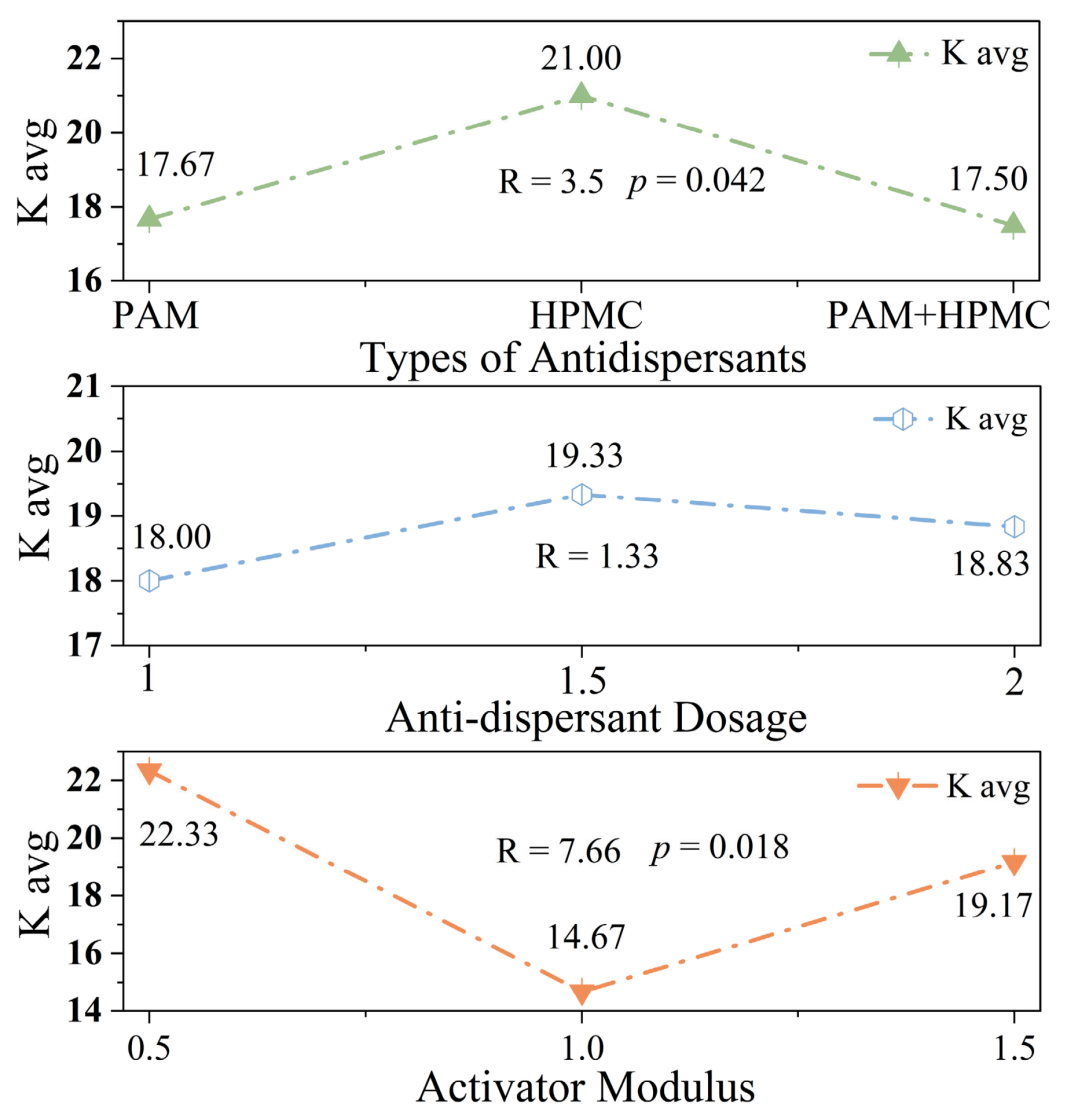
Range analysis of initial setting time in alkali-activated slag slurry.

The results of the range analysis for initial setting time are shown in Figure 14. The degree of influence of each factor, from greatest to least, is as follows: activator modulus (R = 7.66) > type of anti-dispersant (R = 3.5) > anti-dispersant dosage (R = 1.33). An analysis of variance was conducted using the anti-dispersant dosage with the smallest range as a dummy error term. The results showed that the *p*-values for both the activator modulus (*p* = 0.018) and the type of anti-dispersant (*p* = 0.042) were less than 0.05, indicating statistical significance. Based on the analysis of variance, the confidence interval corresponding to the optimal combination is (10.77, 15.23) min. Combining the optimal levels of each factor, the alkali-activated slag slurry exhibits the shortest initial setting time when the activator modulus is 1.0, the anti-dispersant dosage is 1%, and a blend of PAM and HPMC is used.

### 3.4. Effect of Antidispersant and Activator on the Compressive Strength of Alkali-Activated Slag

(1) Analysis of the compressive strength of alkali-activated slag

**Figure 15:**
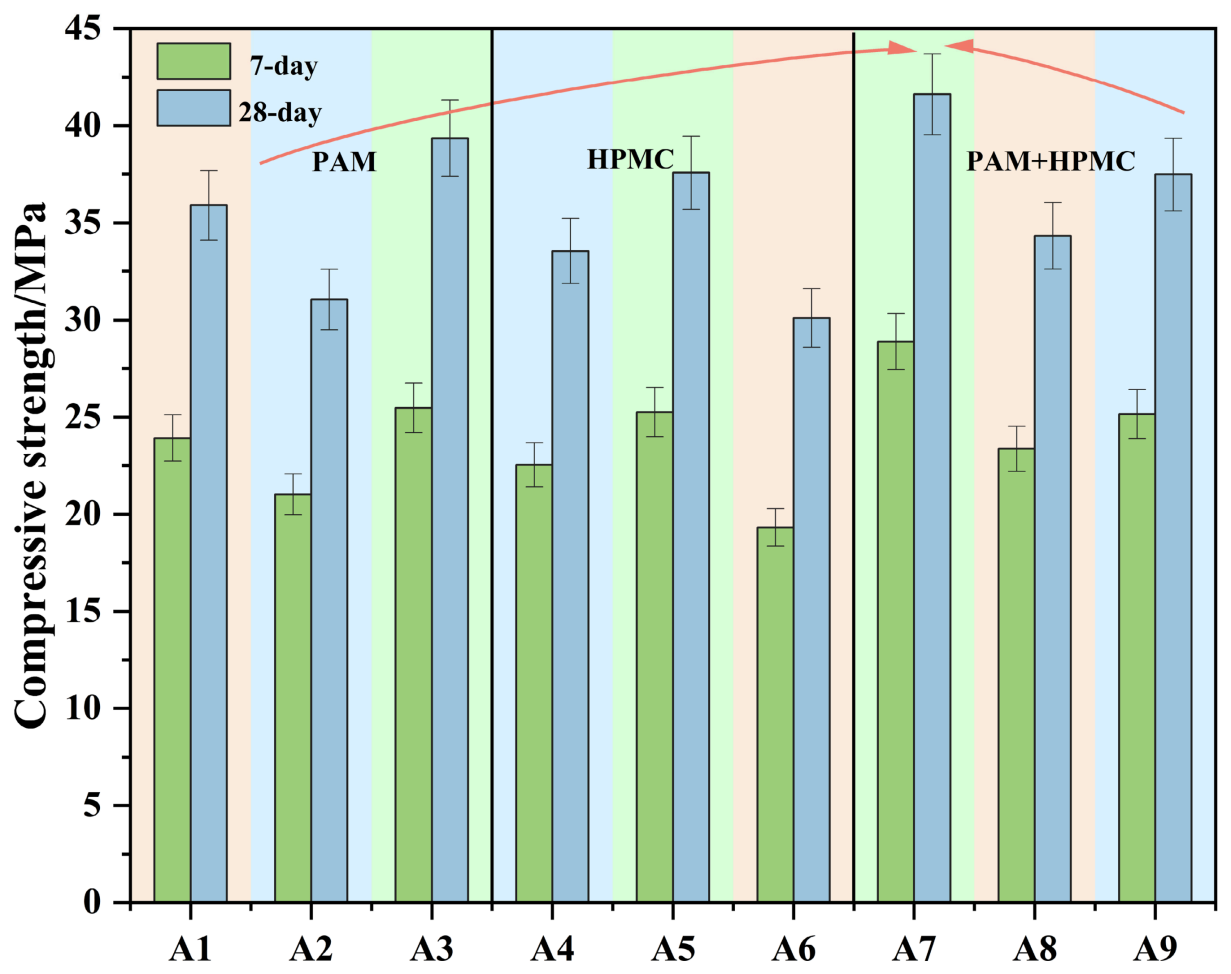
Effect of antidispersant and activator modulus on the compressive strength of alkali-activated slag. The orange, green, and blue backgrounds represent activator moduli of 0.5, 1.0, and 1.5, respectively.

The effects of different types of anti-dispersants, their dosage, and the modulus of the activator on the compressive strength of alkali-activated slag are shown in Figure 15 (the orange, green, and blue backgrounds represent activator moduli of 0.5, 1.0, and 1.5, respectively). The results show that, across all admixture systems, the 28-day compressive strength peaked at a modulus of 1.0, with the optimal groups being A3 (single PAM admixture, 39.36 MPa), A5 (single HPMC admixture, 37.58 MPa), and A7 (blended admixtures, 41.62 MPa). Overall, A7 exhibited the highest strength at all ages, while A6 exhibited the lowest. When the activator modulus was 0.5 or 1.0 and the admixture dosage was the same, the strength of the single-admixture HPMC system was lower than that of the single-admixture PAM or blended admixture systems.

Such performance differences originate from their distinct microscopic working mechanisms. PAM stabilizes suspended slag particles and homogenizes the distribution of hydration products via molecular chain bridging effects, while HPMC mainly regulates the aqueous environment through hydrogen bonding interactions. The combined incorporation of PAM and HPMC integrates the respective advantages of the two polymers, providing a favorable microenvironment for the uniform and dense formation of hydration products and thereby endowing the composite system with superior mechanical performance.

The relatively low compressive strength of alkali-activated slag containing only HPMC is primarily related to its mechanism of action. As a nonionic cellulose ether, HPMC primarily functions to retain water and thicken the slurry [32]; it does not possess flocculation properties on its own and thus struggles to effectively trap fine particles in the slurry. This results in the loss of some solid particles during the underwater molding process, thereby reducing the strength of the hardened product. Furthermore, HPMC exhibits a significant air-entraining effect [33]; the hydrophobic groups on its molecular chains introduce a large number of microbubbles during mixing, which form closed voids after hardening, increasing porosity and reducing density. Therefore, the strength of the system containing only HPMC is inferior to that of systems containing only PAM or those with a combination of HPMC and PAM.

(2) Range analysis of compressive strength

**Figure 16:**
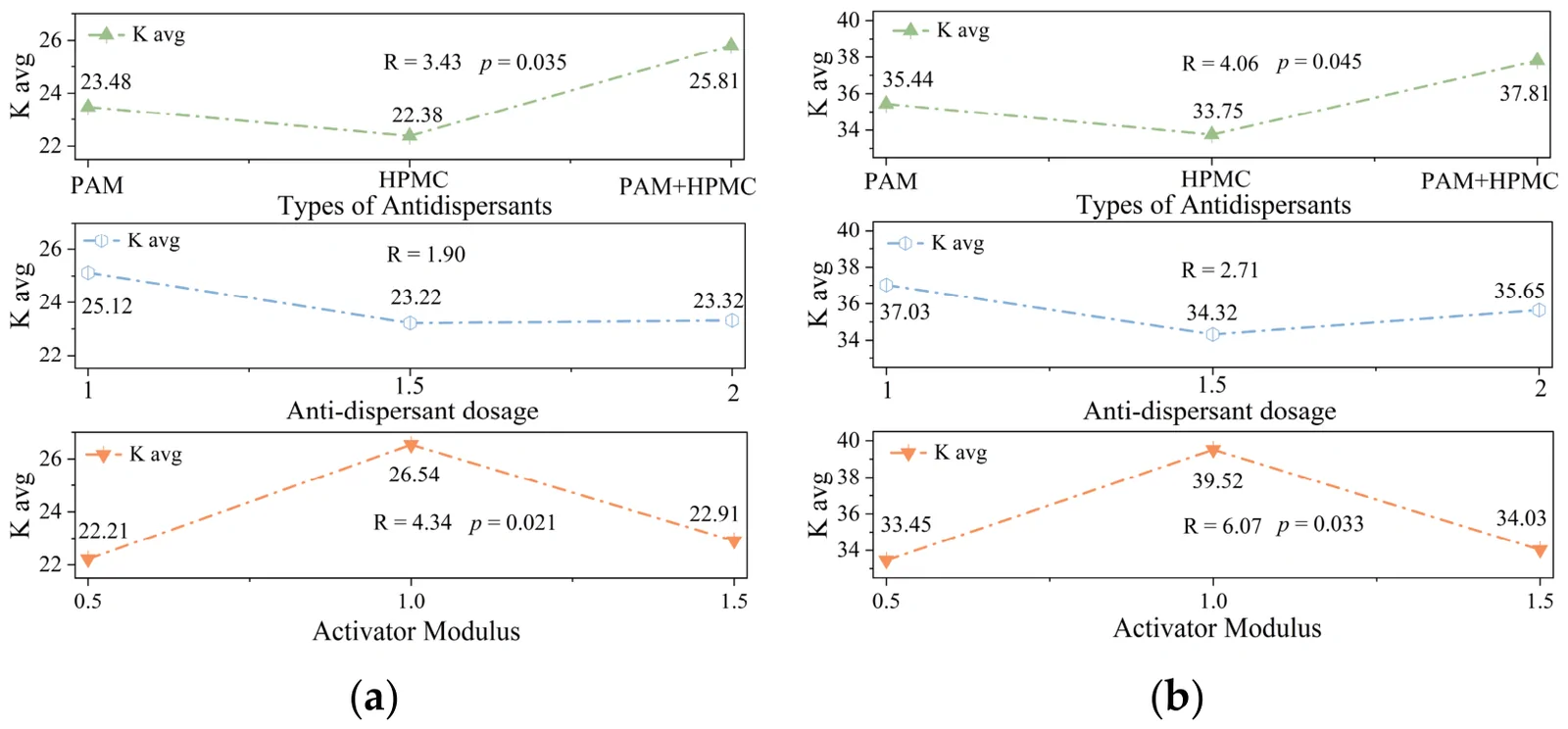
Range analysis of compressive strength of alkali-activated slag at 7 and 28 days. (**a**) Analysis of compressive strength at 7 days. (**b**) Analysis of compressive strength at 28 days.

The results of the range analysis for 7-day and 28-day compressive strengths are shown in Figure 16. The order of influence of each factor on the 7-day compressive strength is: activator modulus (R = 4.34) > type of anti-dispersant (R = 3.43) > anti-dispersant dosage (R = 1.90). For 28-day compressive strength, the order of influence was: activator modulus (R = 6.07) > type of anti-dispersant (R = 4.06) > anti-dispersant dosage (R = 2.71).

An analysis of variance was performed using the anti-dispersant dosage with the smallest range as a dummy error term. The results showed that for the 7-day compressive strength, the *p*-values for the activator modulus (*p* = 0.021) and the type of anti-dispersant (*p* = 0.035) were both less than 0.05; for the 28-day compressive strength, the *p*-values for the activator modulus (*p* = 0.033) and the type of anti-dispersant (*p* = 0.045) were both less than 0.05, reaching statistical significance. Based on the analysis of variance, the confidence intervals corresponding to the optimal combination were (25.20, 32.58) MPa and 28-day compressive strength (38.73, 44.51) MPa. Considering the optimal levels of all factors, the compressive strength of alkali-activated slag reached its maximum when the activator modulus was 1.0, the anti-dispersant dosage was 1%, and a blend of PAM and HPMC was used.

### 3.5. Economic Evaluation and Engineering Applicability

Material cost is a decisive factor limiting the large-scale engineering application of polymer-modified alkali-activated slag slurry. Based on the current market prices of industrial-grade materials (anionic PAM: 5400–8000 RMB/ton; HPMC: 15,500–20,000 RMB/ton), the unit material cost of the 1:1 PAM/HPMC blended system with a total dosage of 1.0% is 104.5–140 RMB per ton of slag. This value is 24.5–86 RMB/ton higher than that of the single PAM system (54–80 RMB/ton slag), but considerably lower than that of the single HPMC system (155–200 RMB/ton slag). Considering the remarkable improvements in anti-dispersion capability and compressive strength, as well as the comprehensive economic benefits brought by reduced solid loss during underwater casting and prolonged service life of structural components, the marginal cost increase in the blended system is economically rational for practical underwater engineering. Consequently, the PAM-HPMC composite system achieves a desirable balance between superior performance and cost viability, exhibiting great potential for practical engineering promotion.

### 3.6. Effect of Anti-Dispersant and Activator on Phase Composition of Alkali-Activated Slag

**Figure 17:**
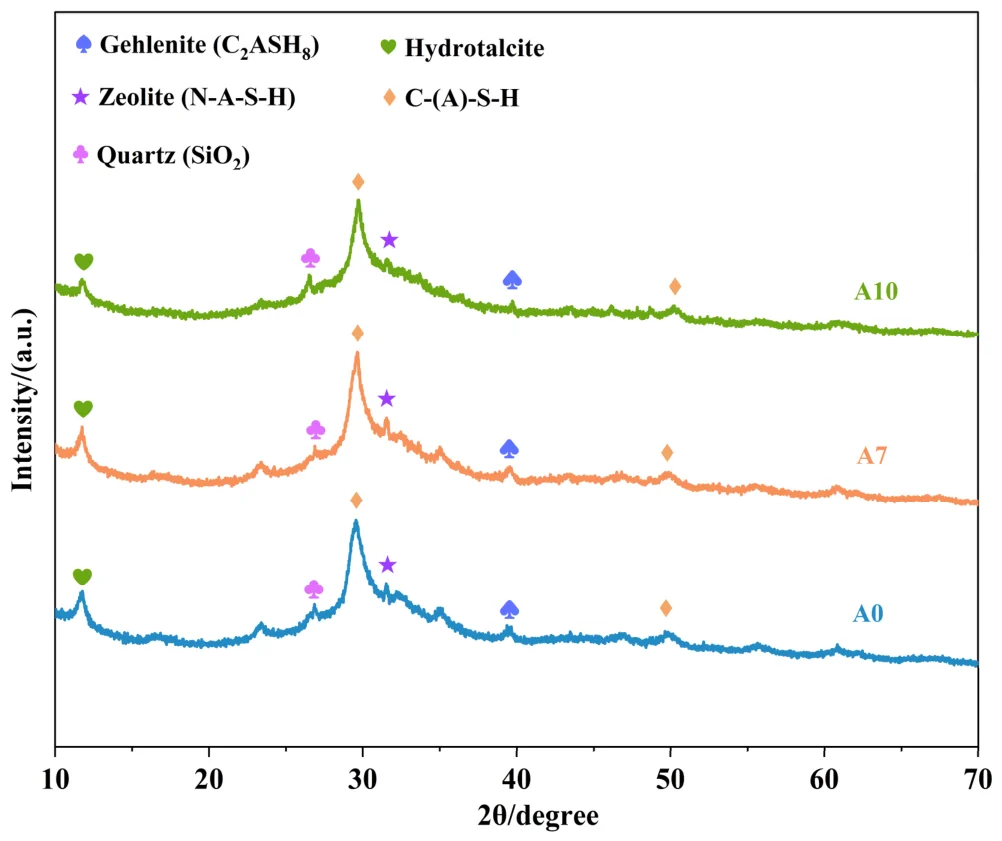
XRD of A0, A7 and A10.

Figure 17 shows the XRD patterns of A0, A7, and A10 after 7 days of curing. Characteristic diffraction peaks of gehlenite (C_2_ASH_8_), zeolite (N-A-S-H), quartz (SiO_2_), hydrotalcite, and the C-(A)-S-H gel were identified in all groups [34,35]. Among these, gehlenite and hydrotalcite are semi-crystalline secondary phases embedded in the gel matrix, while quartz originates from unhydrated slag particles.

Compared with A0, A7 exhibits enhanced diffraction peaks at 29.67° (C-(A)-S-H) and 31.52° (N-A-S-H), while the peak at 26.51° (SiO_2_) is weakened. This indicates that the anti-dispersant is capable of preventing the leaching of Ca^2+^ and Al^3+^ into the aqueous phase, thereby promoting the continuous formation of hydration products.

Compared with A10, A7 exhibits higher diffraction peak intensities at 29.67° and 31.52°, and a lower diffraction peak at 26.51°; furthermore, the hydration peak intensity of A10 is lower than that of A0, while the quartz peak is higher than that of A0. An increase in the activator modulus reduces the alkalinity of the system, delays the depolymerization of the slag glass phase, and results in a significant decrease in the overall degree of hydration. The magnitude of changes in the peak intensities of hydration products caused by changes in the modulus of the activator is greater than that caused by the addition of an anti-dispersant, indicating that the activator has a stronger influence on the slag hydration process than the anti-dispersant.

### 3.7. Effect of Anti-Dispersant and Activator on Molecular Structure of Alkali-Activated Slag

**Figure 18:**
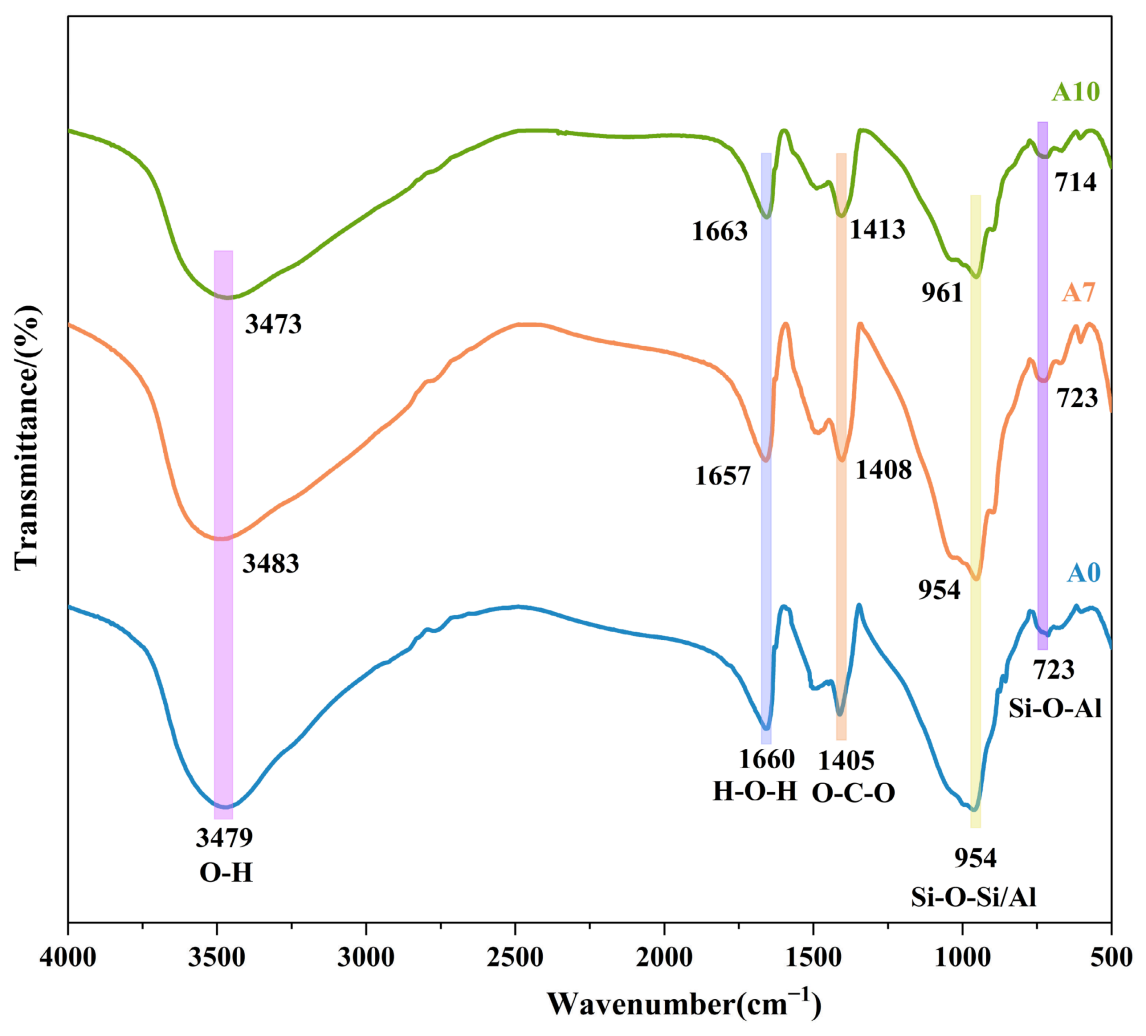
FTIR spectra of A0, A7 and A10.

Figure 18 shows the FT-IR spectra of alkaline slag under the influence of an anti-dispersant and an activator. The three sets of samples exhibit a total of five characteristic absorption peaks: 3479 cm^−1^ corresponds to the O-H stretching vibration of bound water and structural hydroxyl groups; 1660 cm^−1^ corresponds to the H-O-H bending vibration of adsorbed water or gel-bound water; 1405 cm^−1^ corresponds to the asymmetric stretching vibration of O-C-O in surface-layer carbonated carbonates; 954 cm^−1^ corresponds to the Si-O-Si/Al asymmetric stretching vibration in C-(A)-S-H and N-A-S-H gels; and 723 cm^−1^ corresponds to the Si-O-Al bending vibration in secondary phases such as hydrotalcite.

There was no significant shift in the characteristic peak positions of A7 and A0; only the absorption intensity changed slightly, indicating that the addition of the anti-dispersant primarily affected the extent of the alkali-activated slag hydration reaction and did not result in the formation of any significant new phases. The characteristic Si-O-Si/Al peak of A7 is located at 954 cm^−1^, whereas that of A10, which has a higher modulus, shifts to 961 cm^−1^. This is because an increase in the activator’s modulus leads to a higher concentration of soluble SiO_2_ in the solution, which increases the proportion of Si-O-Si structures in the silicate tetrahedra and causes the absorption peak to shift toward higher wavenumbers.

It is worth noting that although the proportion of Si-O-Si bonds in A10 has increased, the absorption intensity of the Si-O-Al vibration is significantly lower than that of A7, and even lower than that of A0. This is because the increase in modulus is accompanied by a decrease in the concentration of alkali in the liquid phase, which inhibits the dissociation of the slag glass matrix. Consequently, the amount of [Al(OH)_4_]^−^ released from the slag decreases, resulting in a reduction in the amount of Al available to participate in the silico-aluminate condensation reaction and form Si-O-Al bonds. This is consistent with the fact that, in the XRD analysis, the diffraction peak intensity of the A10 hydration product was the weakest, while the diffraction signal of the unreacted residual slag quartz was the strongest.

### 3.8. Effect of Antidispersant and Activator on the Microstructure of Alkali-Activated Slag

**Figure 19:**
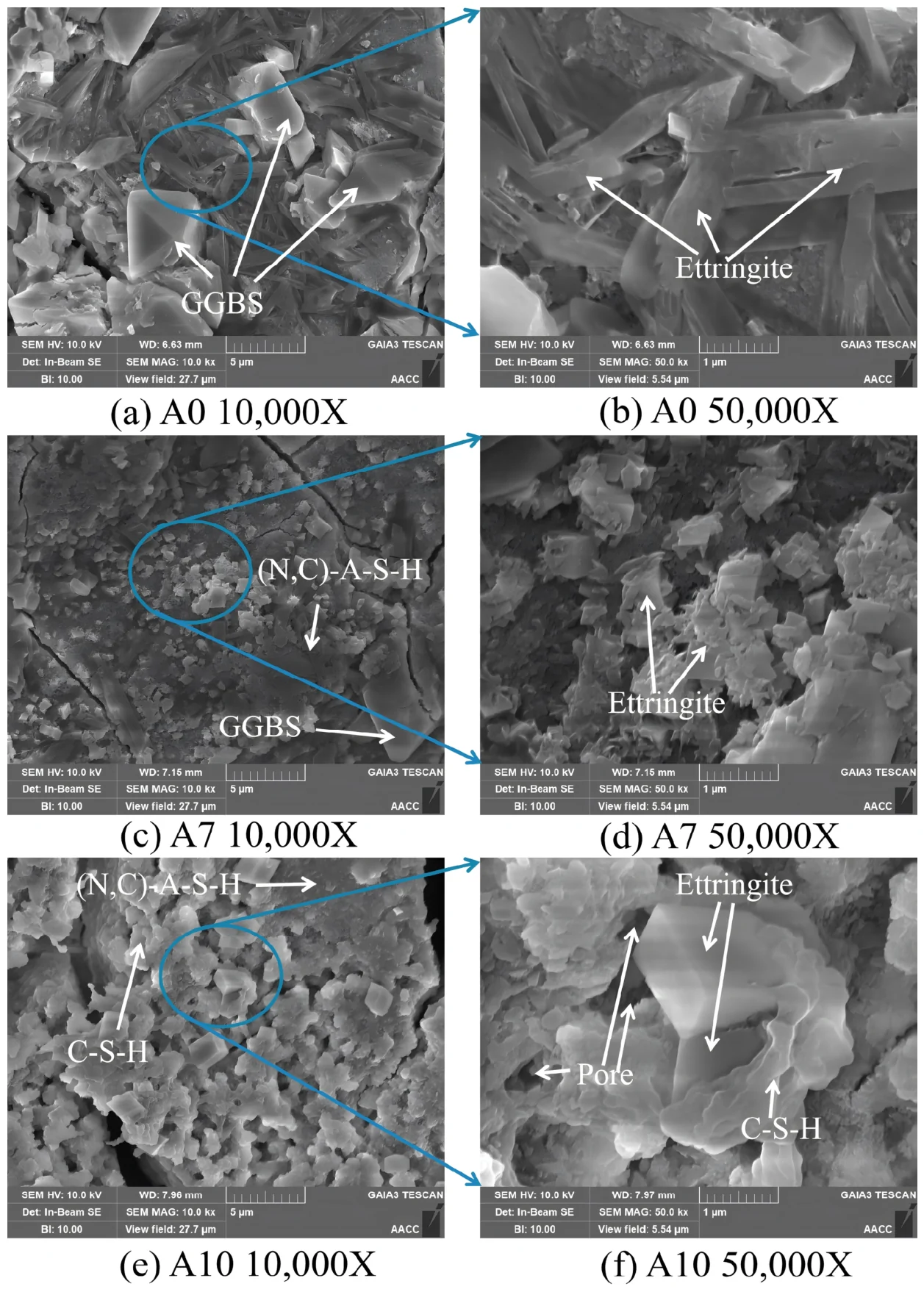
Microscopic morphology of alkali-activated slag.

Figure 19 shows the microstructural characteristics of specimens A0, A7, and A10 after 7 days of curing. Microcracks were observed in all three groups of specimens, primarily resulting from the continuous ingress of external free water in the underwater environment [36]. On the one hand, water penetration increases the local water-to-cement ratio, dilutes the effective concentration of the activator, inhibits the slag-glass reaction, and weakens the consolidation capacity of the hydration products; on the other hand, excess bound water remains trapped during the hardening process, forming a connected pore network and increasing the porosity.

Figure 19a,b show that A0 contains a large number of unreacted slag particles and coarse needle-like crystals, and the C-S-H gel failed to effectively encapsulate these particles and crystalline phase products. The alkali-activated slag slurry without an anti-dispersant exhibits weak retention capacity for active ions. When the slag is exposed to alkali, it rapidly releases ions such as Ca^2+^ and Al^3+^, and these ions tend to migrate outward and leach away in an underwater environment [36], resulting in a decrease in the overall degree of hydration of the specimen. However, ion enrichment and supersaturation occur in localized areas of the slurry, promoting excessive precipitation of crystalline hydration products and leading to coarsening.

Figure 19c,d show that the number of unreacted slag particles in A7 has significantly decreased, and the hydrated crystalline phase products exhibit a fine, flake-like morphology, densely dispersed within a continuous C-S-H and (N,C)-A-S-H gel matrix, forming a dense interfacial structure. Furthermore, the number of coarse, needle-like crystals has decreased dramatically, which may be attributed to the dual regulation of ion leaching behavior and the crystal nucleation environment by the anti-dispersant. HPMC and PAM molecular chains adsorb onto the surface of the slag particles through complexation [19,20], slowing the leaching rates of Ca^2+^ and Al^3+^ while simultaneously inhibiting the loss of active ions to the external aqueous environment. As a result, more active ions are retained within the slurry to participate in the hydration reaction, thereby enhancing the overall degree of hydration; at the same time, the polymer network provides a large number of dispersed nucleation sites, reducing local ion supersaturation and promoting the precipitation of hydration products in a fine, multi-oriented form.

Figure 19e,f show that the hydrated crystalline phase products in A10 are significantly larger than those in A7, exhibiting angular block-like and flake-like morphologies, with C-S-H gel layers stacked around the crystals. Furthermore, the microstructure of this group reveals a large number of pores, insufficient continuity of the gel layers, and overall poor density.

**Figure 20:**
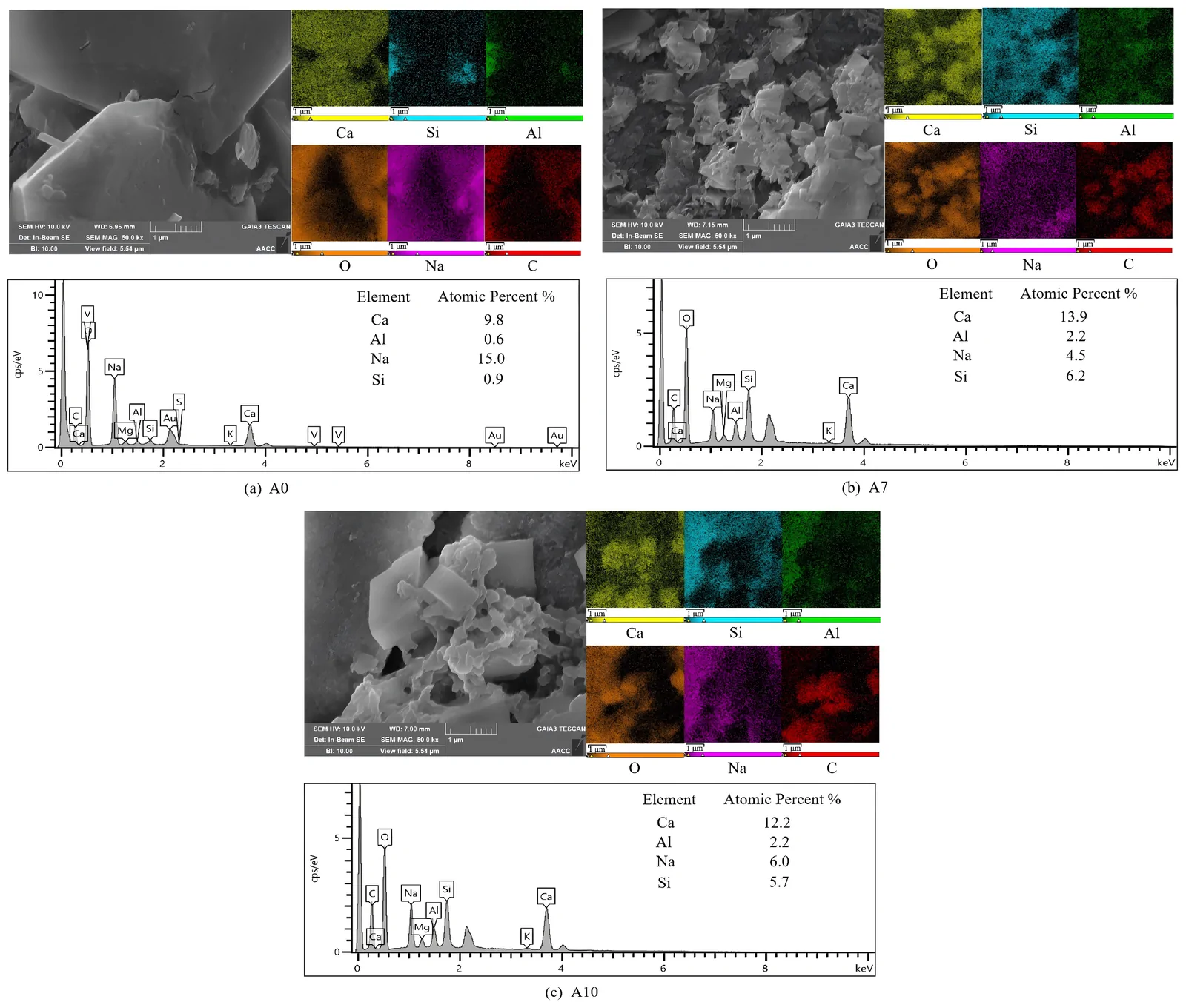
Semi-quantitative EDS mapping analysis of alkali-activated slag. (**a**) Elemental composition of A0; (**b**) elemental composition of A7; (**c**) elemental composition of A10. In the elemental maps, yellow, blue, green, orange, magenta, and red represent Ca, Si, Al, O, Na, and C, respectively.

Figure 20 shows the semi-quantitative EDS mapping analysis results of alkali-activated slag 7 days after hydration. As shown in Figure 20a, the Ca signal is significantly enriched in the A0 block-like region, while the Si and Al signals are relatively weak in the matrix gel region; the Ca/Si, Al/Si, and Na/Si molar ratios are 10.89, 0.67, and 16.67, respectively. These values deviate significantly from the stoichiometric ranges typical of C-S-H, C-A-S-H, and N-A-S-H gels [37], indicating that this region may contain a mixture of unhydrated slag particles and residual crystalline phases of the activator. The substrate region exhibits a low degree of hydration and has not yet formed a continuous gel network.

As shown in Figure 20b, elements such as Ca, Al, Si, and Na are diffusely distributed throughout A7. The molar ratios of Ca/Si, Al/Si, and Na/Si are 2.24, 0.35, and 0.73, respectively. Among these, Al/Si = 0.35 satisfies the aluminum substitution characteristic of C-A-S-H [38]; however, Ca/Si = 2.24 exceeds the typical range for C-A-S-H (0.8–1.4) [39]. The fact that the C-(A)-S-H and N-A-S-H diffraction peaks exhibited the highest intensities in the XRD analysis, and that the main Si-O-Si/Al peak in the FT-IR spectrum was located at 954 cm^−1^ with the greatest absorption intensity, indicates that the gel phase in this region consists primarily of a mixture of C-S-H and N-A-S-H; meanwhile, the relatively high Ca/Si ratio may suggest the presence of a small amount of crystalline calcium phase or unreacted slag particles in this region.

As shown in Figure 20c, the Ca and O signals are significantly enriched in the A10 block-like region, with molar ratios of Ca/Si, Al/Si, and Na/Si of 2.14, 0.39, and 1.05, respectively; the Ca/Si ratio also exceeds the typical range for C-A-S-H. Compared with A7, the elemental distribution in A10 is uneven: the left side is dominated by the gel phase, while the right side has more pores and less gel; overall, its density is lower than that of A7.

### 3.9. Effect of Antidispersant and Activator on the Pore Structure of Alkali-Activated Slag

**Figure 21:**
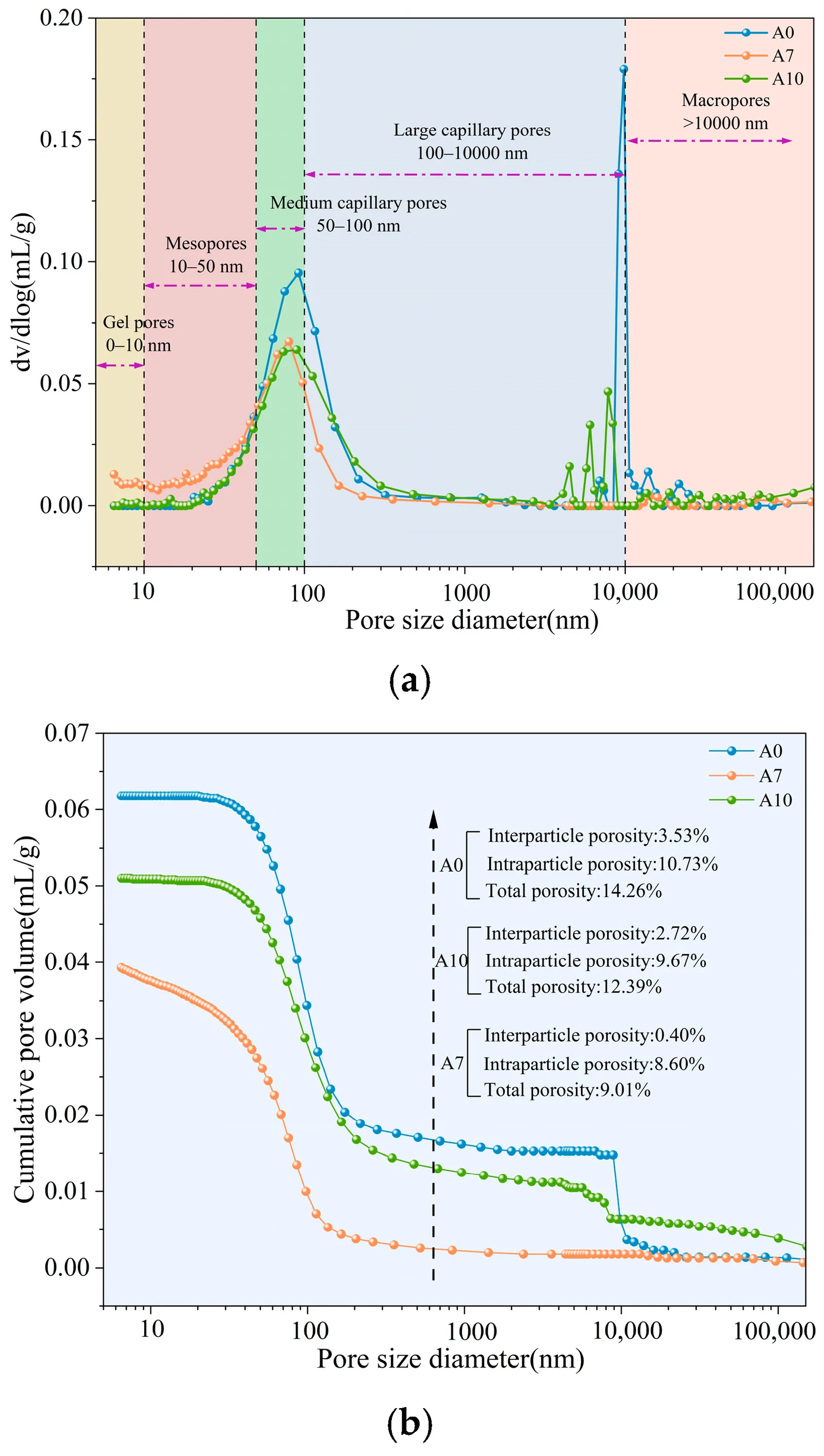
Microscopic pore structure of alkali-activated slag slurry. (**a**) Log differential intrusion curves; (**b**) cumulative pore volume curves.

Figure 21 shows the MIP test results for A0, A7, and A10 after 7 days of hydration. According to pore size classification standards [40,41], pores can be divided into gel pores (0–10 nm), mesopores (10–50 nm), medium capillaries (50–100 nm), large capillaries (100–10,000 nm), and macropores (>10,000 nm). Pores smaller than the average capillary pore size have no significant effect on the compressive strength of alkali-activated slag, whereas large capillaries and macropores are the key pore types that determine the strength of alkali-activated slag; an increase in their volume typically leads to a decrease in strength [42], and they are also the primary harmful pores that impair the material’s mechanical properties and long-term durability [43,44].

As shown in Figure 21a, the volume of A0 in the large capillary and macroporous ranges is significantly higher than that of the A7. Figure 21b also shows that the cumulative pore volume of A0 across the entire pore size range is significantly higher than that of A7. The interparticle porosity and intraparticle porosity of A0 were 3.53% and 10.73%, respectively, while those of A7 decreased to 0.40% and 8.60%, respectively. The reason for the significantly lower interparticle porosity of A7 may be that PAM and HPMC formed a three-dimensional network structure through physical cross-linking of their polymer chains, which, on the one hand, inhibited bubble nucleation and coalescence, and on the other hand, promoted the dense packing of solid particles, thereby effectively reducing the volume of harmful large pores and refining the pore size distribution.

Compared with A7, A10 also exhibited a significant increase in pore volume in the large capillaries and macropores, and its cumulative pore volume was higher than that of A7. The intraparticle porosity of A10 and A7 was 9.67% and 8.60%, respectively, showing a relatively small difference; however, their interparticle porosity was 2.72% and 0.40%, respectively, indicating that the increase in modulus primarily affected the interparticle packing density. As the modulus of the activator increases, the concentration of SiO_3_^2−^ in the solution rises [45], accelerating the formation of initial hydration products on the surfaces of the slag particles. These products preferentially deposit at particle contact points, hindering further particle rearrangement and dense packing. At the same time, a high modulus causes a decrease in the system’s liquid-phase alkalinity, slows down the depolymerization process of the slag-glass matrix, and limits the total amount of hydration products formed, making it difficult to form a continuous and complete gel matrix, which ultimately leads to an increase in the porosity between the particles of the hardened slurry.

## 4. Mechanistic Analysis

### 4.1. Mechanism of Synergy Between PAM and HPMC

**Figure 22:**
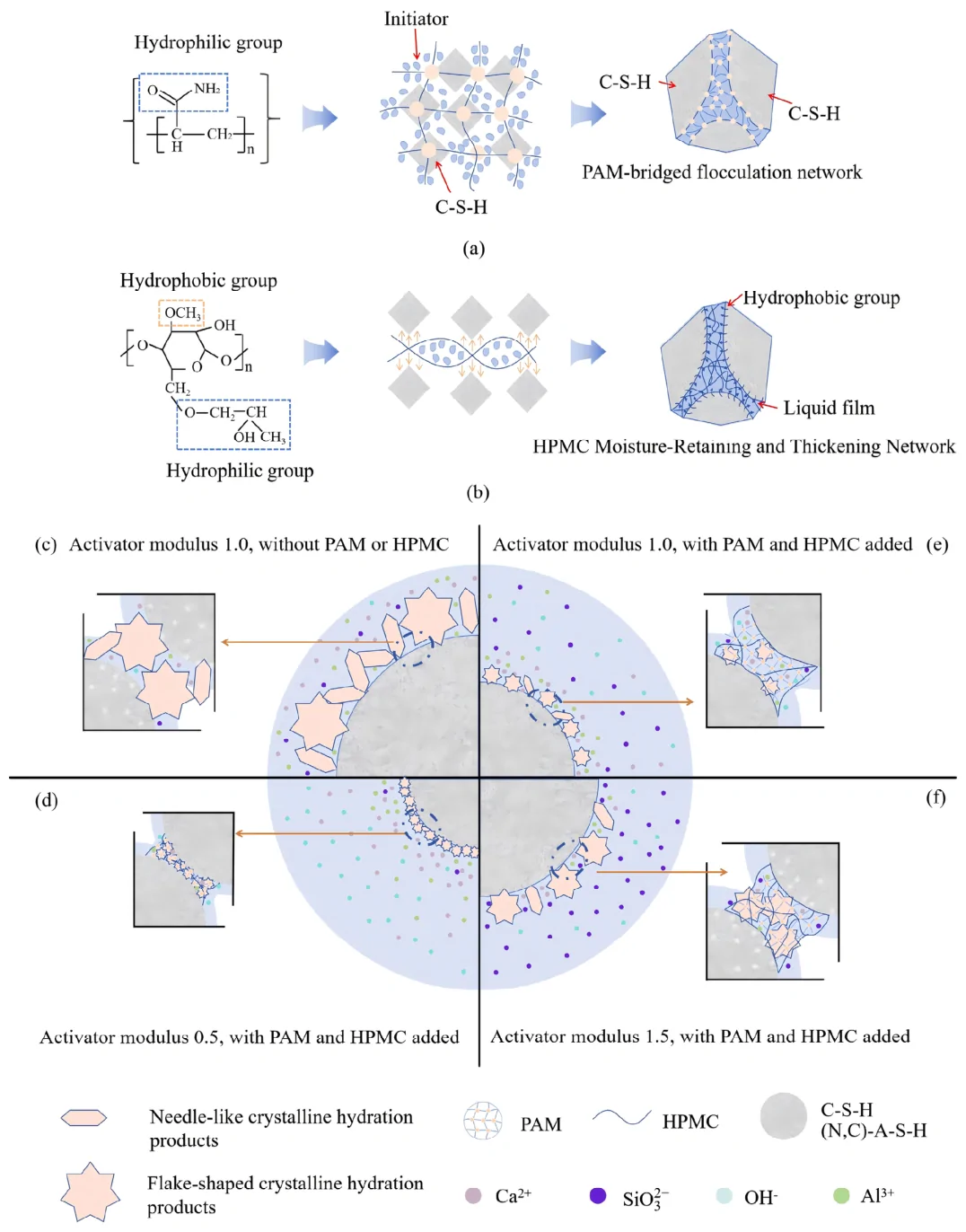
Mechanisms of the antidispersion effects of PAM, HPMC, and excitation agents on modulus. (**a**) Mechanism of PAM’s anti-dispersion effect; (**b**) mechanism of HPMC’s anti-dispersion effect; (**c**) activator modulus 1.0 without PAM or HPMC; (**d**) activator modulus 0.5 with PAM and HPMC added; (**e**) activator modulus 1.0 with PAM and HPMC added; (**f**) activator modulus 1.5 with PAM and HPMC added.

As shown in Figure 22a, PAM molecular chains are rich in amide groups (-CONH_2_), exhibiting strong hydrophilicity; they can adsorb free water through hydrogen bonding [46], causing the molecular chains to swell and increasing the slurry viscosity. At the same time, PAM molecular chains form bridging structures between slag particles, constructing a three-dimensional flocculation network that effectively suppresses particle dispersion and loss in water. Furthermore, the carboxyl groups (-COOH) generated by the hydrolysis of PAM can form complexes with Ca^2+^ and Al^3+^ [47,48], increasing the cross-linking density between molecular chains and forming a gel-like network with stronger cohesive strength.

As shown in Figure 22b, HPMC, as a nonionic cellulose ether, binds free water via hydrogen bonds formed between the hydroxyl groups (-OH) and ether bonds (-O-) on its molecular chains [11], thereby reducing its fluidity and conferring good water retention and dilution resistance to the slurry. HPMC possesses an amphiphilic structure with both hydrophilic and hydrophobic groups [32]; the hydrogen bonds in the hydrophilic groups bind with the activator to form a sol network, while the hydrophobic groups tend to align at the solid–liquid interface, enhancing interparticle adhesion.

After the two materials are blended, the cross-linked flocculation network of PAM and the water-retaining and thickening network of HPMC interweave spatially and complement each other functionally, forming a stable dual-network structure. This structure inhibits the leaching of active ions into the aqueous phase through a dual mechanism of physical encapsulation and chemical complexation, and delays the excessive dilution of the slurry by external water, thereby suppressing the coarse growth of the crystalline phase. This synergistic mechanism enhances the slurry’s resistance to dispersion while maximizing its flowability.

### 4.2. The Essence of Regulating the Activator

As shown in Figure 22c, when the activator modulus is 1.0 and no anti-dispersant is added, the slag continuously releases Ca^2+^ and Al^3+^, promoting the formation of C-S-H and C-A-S-H gels. Due to the slurry’s weak ability to retain active ions, some ions leach out in the underwater environment, while ion supersaturation occurs in certain areas, promoting the free nucleation and continuous growth of crystalline hydration products, resulting in needle-like and plate-like crystalline hydration products. There are a large number of unhydrated slag particles, which have weak bonding with the gel, resulting in a relatively loose overall structure.

As shown in Figure 22d, when the activator modulus is 0.5 and an anti-dispersant is co-blended, the alkalinity of the system increases significantly, accelerating the leaching of Ca^2+^ and Al^3+^, which promotes the formation of C-S-H gel. However, at low modulus values, the concentration of soluble silicate ions is limited, which restricts the total amount of C-S-H gel that can be formed [48]; the early hydration reaction is vigorous, and fine crystalline hydration products densely adhere to and interpenetrate the surface of the C-S-H gel, forming a relatively dense structure, but with limited flowability.

As shown in Figure 22e, when the activator modulus is 1.0 and an anti-dispersant is co-doped, the slag undergoes thorough depolymerization; the anti-dispersant regulates the release of Ca^2+^ and Al^3+^, thereby inhibiting the excessive growth of crystalline hydration products crystals [49], resulting in flake-like products. At the same time, an adequate amount of soluble silicate ions promotes the continuous formation and uniform distribution of C-S-H and C-A-S-H gels, resulting in a dense structure.

As shown in Figure 22f, when the activator modulus is 1.5 and a complex dispersant is co-doped, the soluble silicate ions in the activator preferentially react with the leached Ca^2+^ and Al^3+^, consuming a portion of the Ca^2+^ and reducing the concentration available for crystal phase nucleation, thereby inhibiting the nucleation density and the number of crystal nuclei formed. However, at the limited nucleation sites, the remaining crystal nuclei continue to grow, resulting in excessively large hydration products in the crystalline phase. At the same time, the high modulus reduces the alkalinity of the system, delaying the depolymerization of the slag glass phase. This leads to a decrease in the formation rate of the early gel phase and a lack of continuity, causing an increase in the porosity of the hardened slurry and a reduction in its density.

## 5. Conclusions

This study systematically investigated the synergistic regulation mechanisms of activators and anti-dispersants on the anti-dispersion properties, rheological behavior, initial setting time, and mechanical properties of alkali-activated slag slurries. It clarified the order of importance of each factor’s influence on slurry performance, revealed the evolution characteristics and microscopic mechanisms of slag slurry performance, and provided a basis and reference for the optimization of mix designs and engineering applications of underwater alkali-activated cementitious materials. The main conclusions are as follows:

(1) The activator is the primary factor regulating the anti-dispersion properties, rheological behavior, and compressive strength of alkali-activated slag slurries. When the activator modulus is increased from 0.5 to 1.5, the slurry’s flowability continues to increase, while the yield stress and plastic viscosity decrease significantly; however, the slurry’s resistance to dispersion and compressive strength reach their optimal values at a modulus of 1.0, indicating that the activator’s regulatory effects on the slurry’s fresh workability and mechanical properties are not synchronized.
(2) PAM and HPMC exhibit a synergistic effect in alkali-activated slag slurry. PAM forms a flocculation network through molecular chain bridging, enhancing interparticle cohesion and solid-phase retention capacity; HPMC binds free water through its water-retention properties. The co-blending of the two can simultaneously improve the slurry’s resistance to dispersion and flowability under low modulus conditions, with the improvement in anti-dispersion performance exceeding that of single-additive systems.
(3) A blend of HPMC and PAM can regulate the dissolution rates of Ca^2+^ and Al^3+^, reduce the loss of active ions in underwater environments, and inhibit excessive coarsening of crystalline hydration products; at the same time, it provides a large number of dispersed nucleation sites, promoting the continuous and uniform distribution of C-S-H and (N,C)-A-S-H gels, thereby forming a dense interfacial structure.
(4) Taking multiple factors into account, when the activator modulus is 1.0, the total dispersant content is 1%, and PAM and HPMC are blended, the alkali-activated slag slurry achieves an optimal balance between dispersion resistance and flowability.

This study employed an L_9_(3^3^) orthogonal experimental design; however, with only two degrees of freedom for error, the experimental precision is somewhat limited. Furthermore, by fixing the dosage of liquid sodium silicate and adjusting the amount of NaOH to modulate the activator modulus, differences in total Na_2_O content arose among the test groups; therefore, the activator modulus referred to in this paper represents the comprehensive effect of the activator. Furthermore, this study fixed the test water temperature and the amount of silicon source added, which differs from the complex marine environment. Finally, this study only investigated the fresh slurry properties and early-age mechanical properties of this alkali-activated slag; systematic research on long-term durability, engineering suitability, and economic benefits has not yet been conducted.

In the future, multi-gradient marine environment coupling experiments will be conducted to establish a correlation between laboratory data and actual marine conditions. Concurrently, the behavior of total alkali content will be clarified, long-term service performance will be evaluated, and costs will be optimized, thereby providing a basis for the engineering application of alkali-activated cementitious materials in underwater environments.

## Figures and Tables

**Table 1:** Chemical composition of GGBS (wt%).

Component	SiO_2_	CaO	A1_2_O_3_	Fe_2_O_3_	MgO	Na_2_O	MnO	K_2_O	Others	Loss
Content	31.35	34.65	18.65	0.57	9.31	0.61	0.41	1.12	2.63	0.70

**Table 2:** Mix proportions of alkali-activated slag slurry (per 400 g of GGBS).

Label	GGBS (g)	Sodium Silicate (g)	NaOH (g)	Anti- Dispersant (g)	Dosage (wt%)	Modulus M	Water (g)
A1	400	55.5	39.6	PAM	1.0	0.5	145.3
A2	400	118.8	16.3	PAM	1.5	1.5	105.7
A3	400	92.4	26.0	PAM	2.0	1.0	122.2
A4	400	118.8	16.3	HPMC	1.0	1.5	105.7
A5	400	92.4	26.0	HPMC	1.5	1.0	122.2
A6	400	55.5	39.6	HPMC	2.0	0.5	145.3
A7	400	92.4	26.0	PAM + HPMC	1.0	1.0	122.2
A8	400	55.5	39.6	PAM + HPMC	1.5	0.5	145.3
A9	400	118.8	16.3	PAM + HPMC	2.0	1.5	105.7

**Table 3:** Yield stress and plastic viscosity of alkali-activated slag slurry under different mix proportions.

Label	A1	A2	A3	A4	A5	A6	A7	A8	A9
Yield Stress (Pa)	16.82	6.61	18.97	4.41	20.71	10.91	10.42	17.92	19.91
Plastic Viscosity (Pa·s)	9.06	1.28	5.40	3.26	6.51	5.71	1.23	8.81	7.09

## Data Availability

The original contributions presented in the study are included in the article, further inquiries can be directed to the corresponding authors.

## Abbreviation

The definition of terms.

PAM	Polyacrylamide
HPMC	Hydroxypropyl methylcellulose
GGBS	Granulated blast furnace slag
